# Impairing cardiac oxygen supply in swimming coho salmon compromises their heart function and tolerance to acute warming

**DOI:** 10.1038/s41598-023-47713-5

**Published:** 2023-12-01

**Authors:** Andreas Ekström, Brian Hendriks, Jacey C. Van Wert, Matthew J. H. Gilbert, Anthony P. Farrell, Steven J. Cooke, David A. Patterson, Scott G. Hinch, Erika J. Eliason

**Affiliations:** 1 Department of Biological and Environmental Sciences, University of Gothenburg, PO Box 463, 40530 Gothenburg, Sweden; 2 Pacific Salmon Ecology and Conservation Laboratory, Department of Forest and Conservation Sciences, The University of British Columbia, Vancouver, BC V6T 1Z4 Canada; 3 Department of Ecology, Evolution and Marine Biology, University of California, Santa Barbara, CA 93106-9620 USA; 4 Department of Zoology, The University of British Columbia, Vancouver, BC V6T 1Z4 Canada; 5 Fish Ecology and Conservation Physiology Laboratory, Department of Biology, Carleton University, Ottawa, ON K1S 5B6 Canada; 6 Fisheries and Oceans Canada, Cooperative Resource Management Institute, School of Resource and Environmental Management, Simon Fraser University, Burnaby, BC V5A 1S6 Canada

**Keywords:** Ecophysiology, Zoology, Cardiovascular biology, Circulation, Metabolism, Respiration

## Abstract

Climatic warming elevates mortality for many salmonid populations during their physically challenging up-river spawning migrations, yet, the mechanisms underlying the increased mortality remain elusive. One hypothesis posits that a cardiac oxygen insufficiency impairs the heart’s capacity to pump sufficient oxygen to body tissues to sustain up-river swimming, especially in warm water when oxygen availability declines and cardiac and whole-animal oxygen demand increases. We tested this hypothesis by measuring cardiac and metabolic (cardiorespiratory) performance, and assessing the upper thermal tolerance of coho salmon (*Oncorhynchus kisutch*) during sustained swimming and acute warming. By surgically ligating the coronary artery, which naturally accumulates arteriosclerotic lesions in migrating salmon, we partially impaired oxygen supply to the heart. Coronary ligation caused drastic cardiac impairment during swimming, even at benign temperatures, and substantially constrained cardiorespiratory performance during swimming and progressive warming compared to sham-operated control fish. Furthermore, upper thermal tolerance during swimming was markedly reduced (by 4.4 °C) following ligation. While the cardiorespiratory capacity of female salmon was generally lower at higher temperatures compared to males, upper thermal tolerance during swimming was similar between sexes within treatment groups. Cardiac oxygen supply is a crucial determinant for the migratory capacity of salmon facing climatic environmental warming.

## Introduction

For many salmonids (*Salmonidae spp.*), reproduction and life-time fitness depend on a challenging up-river migration to a natal spawning area. Some *en-route* mortality is common even during normal, benign river conditions, however, increasing river temperatures associated with climate warming are being associated with higher migration failure and mortality among many salmon populations^1–6^. Moreover, en-route mortality in female salmon can be several fold higher compared to males during stressful migration conditions^7^, threatening the persistence of salmon populations. Clearly, understanding the mechanisms for the increased mortality and migration failure is an immediate conservation concern given salmon’s immense ecological, cultural and economic value worldwide.

Increased migration failure is likely related to constraints in the physiological performances that govern prolonged, high-intensity swimming^3,8–10^. The physiological potential of salmon to reach their spawning grounds is largely determined by their aerobic metabolic capacity^3^. In turn, the heart must pump an adequate supply of blood to body tissues to deliver oxygen and nutrients essential for cellular metabolic processes during migration. Indeed, the warming-induced mortality of migratory salmonids has been linked to a reduced aerobic metabolic scope due to failing cardiac performance at supra-optimal temperatures^3,8,9^. Moreover, in sockeye salmon (*Oncorhynchus nerka*), fatigue during swimming was linked to an inability to elevate cardiac output (i.e., amount of blood pumped by the heart per unit time) at elevated temperatures^10^. However, the mechanism for this type of cardiac failure is still a matter of speculation. One working hypothesis postulates that an oxygen limitation to cardiac tissues performing high workloads at elevated temperatures may cause cardiac failure^8,11–13^.

This hypothesis is founded on the vertebrate heart being a highly aerobic tissue that depends on a continuous oxygen supply for energy production to sustain cardiac function^14^. Salmonid hearts have two oxygen supply routes. Like mammals, they have a coronary artery, which delivers well-oxygenated arterial blood directly from the gills to the outer compact myocardial layer of the heart^15–17^. But, the outer compact myocardium represents only ~ 20–40% of ventricular mass, and the remainder comprises inner spongy myocardium which extracts the oxygen remaining in the venous blood (the luminal oxygen supply) returning to the heart after having delivered oxygen to systemic tissues. The luminal oxygen supply may be considered precarious because the driving pressure for this diffusion is the partial pressure of venous oxygen (P_V_O_2_), which readily decreases with increasing activity and temperature when oxygen extraction by systemic tissues increases^11,12,18,19^. Nonetheless, the flow rate of the venous oxygen supply during swimming increases because cardiac output increases. Coronary blood flow similarly increases with swimming and temperature in salmonids, implying its functional importance for the required increase in cardiac performance to support swimming activity^20,21^. However, during their spawning migrations Pacific salmonids develop vascular lesions and plaques in the coronary artery (termed arteriosclerosis) that can severely occlude the artery and could theoretically decrease coronary blood flow^22,23^. Experimental blockade of the coronary artery by surgical ligation impairs cardiac performances (e.g., stroke volume, cardiac output and ventral aortic blood pressure generation) during swimming or exposure to acute environmental hypoxia or warming^19,21,24,25^. Moreover, coronary ligation reduces aerobic metabolic scope and heart rate scope in rainbow trout (*O. mykiss*)^26^ and lowers the maximum sustained swimming speed (U_crit_) in Chinook salmon (*O. tshawytscha*)^27^. Coronary ligation also lowers the critical thermal maximum (CT_max_; the temperature at which the fish loses equilibrium) in resting rainbow trout^21,24,25^. Consequently, the relevance of the coronary oxygen supply needs to be evaluated with the combined stresses of warming and intense swimming to mimic the migration conditions experienced by salmon today and in the future. Perhaps, the higher mortality of egg-bearing migrating female salmon relates to a lower capacity for increasing coronary blood flow during warming as observed in farmed rainbow trout^21^. This possibility requires a sex-specific examination of the importance of coronary circulation, which remains unexplored in fish.

We tested the hypothesis that the upper thermal tolerance and maximum cardiorespiratory performance during sustained swimming of coho salmon (*Oncorhynchus kisutch,* Walbaum 1792) was dependent on its coronary oxygen supply. We therefore compared fish with an occluded (ligated) coronary artery to sham-operated fish (leaving coronary blood flow intact), while measuring in vivo cardiac performance, oxygen uptake and blood oxygen and metabolite status during sustained swimming. Fish swam steadily at ~ 60% of their U_crit_ in a swim respirometer while being progressively warmed to a temperature at which they fatigued, termed the functional thermal maximum, FT_max_. We predicted that surgical ligation of the coronary artery would reduce FT_max_ relative to sham-operated fish because of impairment of cardiorespiratory capacities. Our secondary objective examined whether sex-specific differences exist in the assessed performances. We predicted that, compared to males, coronary ligation would have greater negative impacts on cardiorespiratory performance in females and that females would have lower FT_max_. Analyses of cardiac and muscle tissue enzymatic activities were also performed to evaluate whether coronary ligation yielded any changes in aerobic (citrate synthase, CS) or anaerobic (lactate dehydrogenase, LDH) cellular metabolism that could be related to cardiorespiratory or animal performance.

## Materials and methods

This study was approved (ethical permit #A17-0160) by the University of British Columbia Animal Care Committee (ACC) in accordance with the Canadian Council on Animal Care. All experiments were performed in accordance with relevant guidelines (including ARRIVE guidelines) and regulations.

### Fish collection and holding

Maturing adult coho salmon (hatchery origin and of mixed sexes and ages; n = 30, see Table 1 for morphometrics) that had recently completed their spawning migration from the sea, were dip-netted at the Chilliwack River Hatchery on September 30th, October 7th and October 24th, 2019. The daily average water temperature of this time was ~ 15.5 °C and was comparable to the historic average (1950–2018) of 15 °C (Fraser River Ewatch, 2021, Government of Canada). Only silver fish (i.e., not fully reproductively mature, gonads still developing and secondary sexual colouration still in progress) were chosen to standardize the maturation level of the fish. The fish were transported ~ 20 km by truck in holding tanks (2700 L, stocking density ≤ 15 fish, > 90% water air saturation) to the Fisheries and Oceans Canada, Cultus Lake Salmon Laboratory in Cultus Lake, British Columbia, Canada where they were transferred to large holding tanks (5.3 m diameter, 8000 L; stocking density ≤ 11 fish) supplied with a flow-through, sand and UV filtered freshwater from the nearby Cultus Lake. The water was aerated to maintain DO > 90% air saturation and water temperature was maintained at 11–12 °C by mixing cold deep lake water with warmer shallow water. A submersed water pump created a rotational water current in each tank for fish orientation. The holding tanks were situated outside, and transparent windows on each tank allowed for a natural diurnal day:night cycle during the holding period. Fish were held at a minimum of ~ 1 day and a maximum of 38 days prior to experimentation and were not fed because they naturally cease feeding during their river migration.

**Table 1:** Morphological characteristics for the coho salmon (*Oncorhynchus kisutch*) in the two test groups.

	Sham-operated			Coronary-ligated		
	Pooled	Male	Female	Pooled	Male	Female
Sample size (n)	16	8	8	14	7	7
Body mass (g)	2070 ± 115	1989 ± 147	2151 ± 181	1819 ± 169	2034 ± 283	1604 ± 169
Body (fork) length (mm)	569 ± 12	561 ± 18	577 ± 15	547 ± 15	567 ± 26	526 ± 15
Condition factor	1.5 ± 0.03	1.4 ± 0.05	1.5 ± 0.04	1.5 ± 0.03	1.5 ± 0.03	1.5 ± 0.05
Relative ventricle mass (%)	0.17 ± 0.004	0.18 ± 0.05	0.16 ± 0.04^#^	0.19 ± 0.005*	0.2 ± 0.08	0.18 ± 0.06
Percent compact myocardium (%)	27.0 ± 1.0	27.0 ± 1.4	27.0 ± 1.6	28.2 ± 1.4	27.2 ± 1.7	29.7 ± 2.6
Relative spleen mass (%)	0.13 ± 0.01	0.15 ± 0.01	0.11 ± 0.01^#^	0.12 ± 0.01	0.15 ± 0.02	0.10 ± 0.01^#^
Relative liver mass (%)	1.6 ± 0.13	1.12 ± 0.02	2.05 ± 0.10^#^	1.63 ± 0.12	1.2 ± 0.04	2.0 ± 0.09^#^
Relative gonad mass (%)	10.8 ± 1.5	5.3 ± 0.5	16.3 ± 0.6^#^	9.2 ± 1.2	5.5 ± 0.5	12.9 ± 1.1^#^

Asterisks (*) denote statistically significant (P < 0.05) differences between treatment groups, and hash signs (#) denote significant differences between sexes within groups.

### Surgical interventions

The surgical interventions were performed during the evening the day before the experimental protocol. Fish were anaesthetized in 12 °C water containing MS-222 (Tricaine methanesulfonate, 150 mg kg^−1^, buffered with NaHCO_3_, 300 mg kg^−1^) before measuring body mass and length, and placing the fish ventral side up on wet foam on a surgery table. Surgical anaesthesia was maintained by continuously irrigating the gills with 12 °C water containing a lower dose of MS-222 (75 mg kg^−1^, buffered with NaHCO_3_, 150 mg kg^−1^). Fish were randomly assigned into two different treatment groups, both of which received a small incision in the *isthmus*^21,27^. For the “coronary-ligated” group, the coronary artery was dissected free and was ligated by tying a 6–0 silk suture around the vessel to permanently restrict coronary blood flow to the heart. For the “sham-operated” group, fish underwent the same surgical procedure except that no suture was placed around the coronary artery and coronary blood flow remained unrestricted. All fish were then surgically instrumented with a Transonic blood flow probe (PSL3, Transonic systems Inc., Ithaca, NY, USA) fitted around the ventral aorta to record cardiac output, and a PE-50 cannula filled with saline (0.9% NaCl) containing heparin (150 IU ml^−1^) was inserted into the *sinus venosus* for collections of venous blood for determinations of blood oxygen carrying capacity, plasma metabolite and hormone concentrations, as well as the P_V_O_2_ (see^28^). Care was taken not to damage the pericardium or surrounding vessels and nerves during these procedures. The flow probe was secured to the fish inside of the opercular cavity using 4–0 silk sutures, and the probe lead and cannula were sutured and secured along the side of the fish and along the dorsal ridge anterior to the dorsal fin using 2–0 silk sutures. The probe lead and cannula were tied together using silk sutures to prevent entanglement. The fish was then placed in a transparent tube in a Brett-type swim tunnel respirometer (Length: 1243 mm, Diameter: 254 mm, Volume: 450 L;^29^), which was continuously supplied with mixed lake water at a temperature of ~ 15 °C. Fish were allowed at least 12 h of overnight post-surgical recovery prior to the experimental protocol during which the water flow in the swim tunnel was set at 0.3 bl s^−1^ for orientation rather than swimming purposes. The tank was covered with black plastic drapes and the fish were monitored via a mounted camera to minimize external disturbances.

### Experimental protocol

During the next morning, routine O_2_ consumption rate (MO_2_) and cardiovascular variables (described below) were continuously recorded in undisturbed fish at 15 °C at 0.3 bl s^−1^ for at least 1 h before a 700 μl (also for subsequent samples, see below) routine blood sample was taken using a heparinized syringe. The collected volume of blood was replaced with a similar volume of saline (0.9% NaCl). The swim speed was then gradually increased by 0.27 bl s^−1^ every min up to a swim speed of 1.5 bl s^−1^, which represents ~ 60% of U_crit_ for Chilliwack coho salmon^30^. This swim speed was then sustained during the swim and acute warming protocol. After ~ 12–15 min of steady-state swimming at 1.5 bl s^−1^, two consecutive MO_2_ determinations were recorded, and a blood sample was taken. Water temperature was then raised (shallow lake water was heated in a nearby water reservoir as required) over a 1 h period (4 °C h^−1^) to 19 °C and maintained at this stable temperature for 40 min. After this period at 19 °C, the two consecutive MO_2_ determinations and blood sampling were repeated. Water temperature was then raised in 1 °C increments with a heating rate of 2 °C h^−1^, and after ~ 12–15 min following each stable temperature increment, the two consecutive MO_2_ determinations and blood sampling were repeated. This procedure was repeated until each fish reached its FT_max_, namely the individual temperature at which the fish fatigued and ceased swimming at 1.5 bl s^−1^ and could not immediately resume sustained swimming when attempting to do so. At FT_max_, a blood sample and a MO_2_ determination were taken. The swim speed was lowered to 0.3 bl s^−1^ and temperature to 15 °C (conditions were restored within 5–15 min dependent on FT_max_, except when a fish fatigued at the start temperature of 15 °C) for recovery. Once a lower, steady-state heart rate had been reached, typically within 40–98 min (62 ± 17 min, average ± SD across all fish), the two consecutive MO_2_ determinations and blood sampling were repeated. The fish was then removed from the swim respirometer and euthanized by cranial percussion followed by transection of the spinal cord behind the head and was then dissected to remove the ventricle, liver, spleen, gonads and two muscle samples (~ 0.5 cm thick, containing white and red muscle) that were collected from the side of the fish posterior to the dorsal fin. The ventricle was bisected across the valves to the apex and one half of the ventricle was stored in 70% ethanol for subsequent determinations of the proportions of compact myocardium according to Farrell et al.^31^. The other half of the ventricle and the muscle tissues were freeze clamped in liquid nitrogen and stored at − 80 °C for later analyses. Fish that would not swim properly (n = 1), or fish in which the experimental recording equipment failed during the experiment (n = 3), were excluded from the study.

### Recording of cardiorespiratory variables

MO_2_ was determined by closing the swim tunnel to water inflow for ~ 15 min, which resulted in a 3–5% decline (slope) in the air saturation of the water circulating within the respirometer. The decline in air saturation in the tunnel was recorded using a FireSting fibreoptic O_2_ sensor and temperature sensor connected to a FireSting O_2_ meter (PyroScience, Aachen, Germany) mounted in three-way connectors in an external loop comprising vinyl tubing and a water pump (Eheim universal 1046, Germany) that circulated the water from the swim tunnel. The O_2_ optode was two-point calibrated at 15 °C in fully deoxygenated water (0% air saturation, achieved by adding sodium sulphite to water) and in air-bubbled fully saturated water (100% air saturation). Resuming water inflow restored the air saturation to 100%.

Cardiac output was measured with a Transonic blood flow probe connected to a Transonic blood flow meter (T206, Transonic Systems, Ithaca, NY), and the probe was two-point calibrated prior to each experimental run. All flow probes were calibrated to correct for temperature effects on the flow readings across the thermal range of the experimental protocol, according to the manufacturer’s instructions.

Signals from the recording equipment were relayed to a PowerLab system (ADInstruments, Castle Hill, Australia) connected to a laptop with LabChart Pro software (version 7.3.8; ADInstruments, Castle Hill, Australia).

### Calculations

MO_2_ was calculated from the linear decline (slope) in water air saturation caused by fish respiration (∆%airsat/t) according to: MO_2_ = [(V_r_ − V_f_) * (∆%airsat/t) * α]/M_b_, where V_r_ is the volume of the swim respirometer, V_f_ is the volume of the fish assuming that 1 g equals 1 ml of water, α is the temperature specific solubility coefficient of water O_2_ in freshwater and M_b_ is the body mass of the fish. The background respiration rate in the swim-tunnel was recorded for several hours following every experimental protocol after the fish had been removed. The resultant slope (∆%airsat/t) was subtracted from that acquired from the fish. The MO_2_ was calculated by averaging the two duplicate MO_2_ recordings at each experimental level as outlined above.

Cardiac output, subsequently normalized to body mass, was determined as the average of at least three 20–60 s recordings of continuous blood flow in the ventral aorta, around the timepoint at which MO_2_ was recorded as described above. Heart rate (beats min^−1^) was determined from the pulsatile blood flow recordings, and stroke volume was calculated by dividing cardiac output by heart rate. Arterio-venous O_2_ extraction was calculated as MO_2_ divided by cardiac output.

Fish condition factor was calculated as: (100*body mass)/body length^3^, using body mass in grams and body length in centimetres. Relative ventricle, liver, spleen and gonad mass were calculated by dividing organ mass by body mass. The proportion of compact myocardium was calculated as a percentage by dividing compact myocardial dry mass by the total ventricular (i.e., spongy and compact masses) dry mass.

### Blood oxygen carrying capacity and plasma analyses

P_V_O_2_ was determined by injecting a 300 ml sub-sample of each blood sample into a chamber with an integrated two-point calibrated (see above) FireSting fibreoptic O_2_ sensor connected to a FireSting O_2_ meter (PyroScience, Aachen, Germany). The chamber was sealed and placed into a separate (from the fish) undisturbed section of the swim respirometer, which maintained the correct ambient water temperature as it changed during the protocol. A value for P_V_O_2_ was taken after the signal had reached a plateau which typically occurred within ~ 3 min. The blood was then re-pooled in an Eppendorf tube with the original sample for subsequent analytical procedures. Haematocrit was determined as the average fractional erythrocyte to plasma volume after centrifugation of two microcapillary tube samples centrifuged at 10,000 rpm for 5 min. Haemoglobin concentration ([Haemoglobin]) was determined with a handheld Hb 201 + meter (Hemocue AB, Ängelholm, Sweden), and values were corrected for fish blood according to Clark et al.^32^. The mean corpuscular [haemoglobin] (MCHC) was calculated as MCHC = Haemoglobin/Haematocrit*100. The remaining blood was centrifuged at 7000 rpm for 5 min to separate the plasma, which was frozen in liquid nitrogen and stored at − 80 °C for subsequent analysis of plasma [lactate] and [glucose] using a glucose and L-lactate analyzer (YSI 2300 stat plus, USA) according to Farrell et al.^33^. Plasma [Na^+^] and [K^+^] were determined using an XP Five-channel Flame Photometer (BWB Technologies, UK), and plasma [cortisol] was determined using a cortisol ELISA kit from (Neogen, USA) according to manufacturer’s instructions. All measurements on plasma samples were run in duplicate and averaged.

### Tissue metabolite and enzyme analyses

The [lactate] in cardiac and white muscle tissues were determined in triplicate homogenates. Frozen samples were ground in liquid nitrogen and ~ 20 mg of sample was treated with ice-cold 8% HClO_4_ and sonicated on ice. The homogenate was centrifuged at 10,000*g* for 10 min at 4 °C. The supernatant was neutralized using 3 M K_2_CO_3_ and was centrifuged at 10,000*g* for 10 min at 4 °C, and the extracts were subsequently aliquoted and stored at -80 °C until further analyses. Thawed samples were run on a FLUOstar Omega multi-mode microplate reader (BMG Labtech, Germany) using lactate dehydrogenase to catalyse the oxidation of lactate with the reduction of NAD + at 340 nm and a lactate standard curve to measure [lactate]^34^.

The activities of lactate dehydrogenase (LDH) and citrate synthase (CS) in cardiac, white and red muscle tissues were determined at 8, 15, 20, 25 and 30 °C according to previously established protocols^35^. Briefly, frozen tissue (~ 25 mg) was homogenized in homogenization buffer (0.1% Triton, 50 mmol l^−1^ HEPES, 1 mmol L^−1^ EDTA, pH 7.4) with 0.5 mm zirconium oxide beads in a bead beater (Fisherbrand Bead Mill 24 Homogenizer) kept at 4 °C for two 30 s cycles and was kept for 1 min on ice between each cycle. The supernatant was then separated, aliquoted (300 μl) and stored at − 80 °C until further analyses. Samples were subsequently read in triplicate on a FLUOstar Omega microplate reader at 340 nm to detect the disappearance of NADH for LDH activity, or 412 nm to measure the production of 5-thio-2-nitrobenzoic acid, a proxy for CS activity. Absorbance readings were normalized using the Pathlength sensor, and activity levels for LDH and CS were calculated with an extinction coefficient of 6.22 and 13.6 mmol^−1^ cm^−1^, respectively.

### Statistics

Statistical analyses were performed using IBM SPSS statistics 28 software, and statistical significance was accepted at *P* ≤ 0.05. Values are presented as mean ± S.E.M. unless otherwise stated. The sample sizes used for the between group comparisons for all variables were deemed appropriate based on the experience of the authors from previous work in similar types of experiments on salmonids, e.g., see^10,24^. Between treatment or sex differences in FT_max_, as well as body or organ morphometrics, was determined by independent t-tests. Normality and homogeneity of variances were determined using Shapiro–Wilk’s and Levene’s tests, respectively. Linear mixed models were used to evaluate differences between treatment groups or sexes in cardiorespiratory, haematological and blood plasma variables at four experimental levels; at routine conditions at 15 °C, when swimming at 15 °C before the temperature ramping, at FT_max_ and following recovery at 15 °C. Experimental level (repeated measures variable), experimental treatment (coronary-ligated vs. sham-operated), sex (male vs. female) and its interactions were modelled. The values during warming were not included in the model due to declining sample sizes at higher temperatures particularly in the coronary-ligated group. Linear mixed models were also used to evaluate differences between treatment groups or sexes regarding the effects of increasing assay temperature (8, 15, 20, 25 and 30 °C, repeated measures variable) on the enzymatic activities of LDH and CS. A first-order autoregressive covariance structure provided the best fit to the data in these analyses of the enzyme data. Significant main effects were explored by Bonferroni corrected pair-wise comparisons across experimental levels or temperatures, and between experimental groups or sexes. Variables not adhering to the assumptions of the tests were log_10_ transformed (Relative gonad mass, MO_2_, cardiac output, heart rate, stroke volume, P_V_O_2_, haematocrit, plasma [cortisol], [glucose] and [lactate]). To determine relationships between different variables, Pearson bivariate correlation analyses were performed between the different FT_max_, cardiorespiratory variables at FT_max_, haematological variables at FT_max_, enzymatic variables at FT_max_ and body and cardiac morphometrics. To acquire values for enzymatic activities for LDH and CS at FT_max_, linear regressions were plotted across the thermal range (i.e., 8–30 °C) and the resulting equations were used to calculate the enzymatic activity at FT_max_ for individual fish.

## Results

Body mass, body length, condition factor, relative liver and gonad masses and the proportion of compact myocardium did not differ between treatment groups (Table 1). While males and females had similar body mass, body length and condition factor, females of both groups had a significantly lower relative spleen mass (T_12_ = − 2.2; *P* = 0.042) and, as expected, higher relative liver (T_12_ = 8.2; *P* < 0.001) and gonad (T_12_ = − 6.6; *P* < 0.001) masses compared to males (Table 1). Relative ventricle mass of the coronary-ligated group was higher than the sham-operated group (T_28_ = − 3.0; *P* = 0.005; Table 1) and was generally lower in females than males across treatment groups (T_28_ = − 2.9; *P* = 0.007). Specifically, sham-operated females had a significantly lower relative ventricle mass relative to males (T_14_ = − 2.9; *P* = 0.012; Table 1), and a similar trend was observed in the coronary-ligated group (T_12_ = − 2.1; *P* = 0.060). The proportions of compact myocardium were not significantly different between sexes in either group.

### Loss of coronary perfusion to the heart impaired FT_max_ in coho salmon

**Figure 1:**
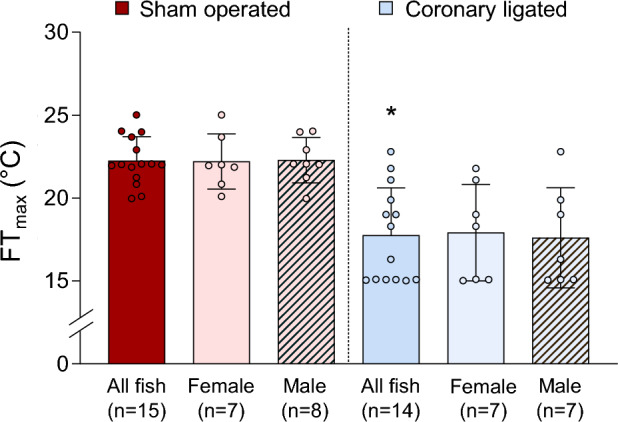
Effects of coronary ligation on the upper thermal tolerance limit in swimming coho salmon (*Oncorhynchus kisutch*). The upper temperature at which fish fatigued (i.e., the functional thermal maximum, FT_max_) during swimming at 1.5 body lengths s^−1^ during an acute thermal challenge (heating rates: 15–19 °C: 4 °C h^−1^; 19 °C- FT_max_: 1 °C h^−1^) in sham-operated and coronary-ligated fish. Data points from individual fish are scattered around each column which represents the average FT_max_ ± SEM. Values for each sex within treatment groups are also depicted, but there were no statistically significant (*P* ≤ 0.05) differences between sexes in either treatment group. Asterisks (*) denote statistically significant differences between treatment groups (sham-operated vs. coronary-ligated), as determined by independent t-tests.

Surgical ligation of the coronary artery impaired the acute warming tolerance of swimming coho salmon, as indicated by a significant (4.4 °C; T_19_ = 5.3; *P* < 0.001) decrease in FT_max_ in the coronary-ligated group (17.8 ± 0.8 °C) relative to the sham-operated group (22.2 ± 0.4 °C, Fig. 1). Furthermore, six individuals of the coronary-ligated group had already reached FT_max_ at the initial temperature of 15 °C, unlike the sham-operated group, in which all fish reached FT_max_ at temperatures > 20 °C. FT_max_ was similar for males and females within groups (coronary-ligated: 17.6 and 17.9 °C, respectively; Sham-operated: 22.3 and 22.2 °C, respectively).

### Impacts of coronary ligation on cardiorespiratory performance during swimming and acute warming in coho salmon

**Figure 2:**
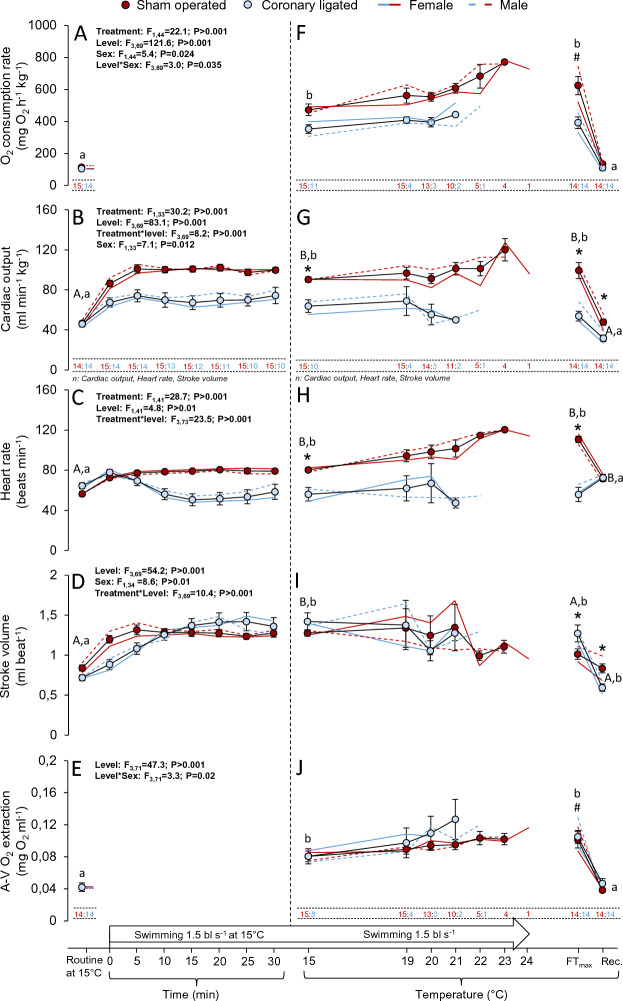
Effects of coronary ligation on cardiorespiratory performances in coho salmon (*Oncorhynchus kisutch*) during sustained swimming and acute warming. Whole animal oxygen consumption rate (**A**,**F**), cardiac output (**B**,**G**), heart rate (**C**,**H**), stroke volume (**D**,**I**) and arterio-venous (**A**–**V**) O_2_ extraction (**E**,**J**) in coronary-ligated (blue symbols) and sham-operated (red symbols) coho salmon during routine conditions (15 °C, 0.3 body lengths s^−1^) or during swimming (1.5 body lengths s^−1^) at 15 °C or during an acute thermal challenge until the critical temperature at which fish fatigued (i.e., the functional thermal maximum, FT_max_; heating rates: 15–19 °C: 4 °C h^−1^; 19 °C- FT_max_: 1 °C h^−1^). The vertical dashed line indicates the onset of the thermal ramping protocol. See specific details for sample sizes (n) below each variable illustration for coronary-ligated (in blue) and sham-operated (in red) groups, n values are shared for heart rate, stroke volume and (**A**–**V**) O_2_ extraction. The data for coronary-ligated or sham-operated female (blue or red solid lines without symbols, respectively) and male (blue and red dashed lines without symbols, respectively) fish are depicted in each panel. The inset tables depict the outcome from the mixed models, which included the data obtained from each fish at four different levels; during routine conditions at 15 °C, swimming at 15 °C, at fatigue (i.e., FT_max_) and following recovery. Only statistically significant (*P* ≤ 0.05) results are displayed here, see Table S1 for full disclosure of the statistical results. Asterisks (*) denote statistically significant differences between treatment groups (sham-operated vs. coronary-ligated) in cases where a significant interaction (Treatment*Level) effect was detected, hash signs (#) denote differences between sexes (male vs. female) across treatment groups (see result section for more details). For variables where a significant interaction between treatment group and experimental level (Treatment*Level) were detected (**B**–**D**), different capital and lower-case letters denote differences between the different levels within sham-operated or coronary-ligated groups, respectively. For variables where no significant Treatment*Level interaction was detected (**A**,**E**), different single lower-case letters denote differences between the different experimental levels across treatment groups. Values are means ± SEM, with the exceptions for the data from males and females, for which only means are depicted for visual clarity.

Neither routine (0.3 bl s^−1^ and 15 °C) MO_2_ nor routine cardiac output was affected by coronary ligation. Routine MO_2_ was 105 ± 10 and 113 ± 7 mg O_2_ h^−1^ kg^−1^ and routine cardiac output was 46 ± 3 and 47 ± 2 ml min^−1^ kg^−1^ in coronary-ligated and sham-operated fish, respectively (Fig. 2A,B; see Table S1 for statistics). Heart rate increased numerically, albeit not significantly (*P* = 0.12) in the coronary-ligated compared to the sham-operated group (64 ± 3 and 56 ± 3 beats min^−1^, respectively). Moreover, stroke volume was slightly but not significantly (*P* = 0.10) reduced in the coronary-ligated group (0.72 ± 0.04 vs. 0.84 ± 0.04 ml min^−1^, respectively; Fig. 2A–D). Routine arterio-venous O_2_ extraction was also similar between groups (Fig. 2E). Thus, routine cardiorespiratory performance was unimpaired by coronary ligation.

Cardiac output initially increased during swimming at 1.5 bl s^−1^ at 15 °C in both groups and plateaued within 5 min. Sham-operated fish elevated cardiac output by gradually increasing both heart rate and stroke volume (Fig. 2B–D). In contrast, 11 of the 14 coronary-ligated individuals experienced a sudden, large decline in heart rate (i.e., bradycardia of ~ 30 beats min^−1^) within the first 5 min of swimming (see Fig. S1). Thus, while heart rate at time-point 0 was matched for both groups (Fig. 2C), the impact of the bradycardia became apparent after 10 min of swimming. Yet, coronary-ligated fish compensated by elevating cardiac output with an increased stroke volume.

After 30 min of swimming heart rate was significantly 31% lower in coronary-ligated compared to sham-operated fish (55 ± 7 and 80 ± 2 beats min^−1^, Fig. 2C). There were no differences between coronary-ligated and sham-operated groups in stroke volume or arterio-venous O_2_ extraction (1.41 ± 0.10 and 1.26 ± 0.04 ml min^−1^, respectively; and 0.078 ± 0.009 and 0.078 ± 0.007, respectively), and both of these variables increased after 30 min of sustained swimming at 15 °C in both groups (Fig. 2D,E). Consequently, after 30 min of swimming, both cardiac output (64 ± 9 and 100 ± 5 ml min^−1^ kg^−1^, respectively) and MO_2_ (342 ± 27 and 463 ± 36 mg O_2_ h^−1^ kg^−1^, respectively) had increased significantly within groups, but were significantly lower in coronary-ligated relative to sham-operated fish by 36 and 26%, respectively (Fig. 2A,B).

Warming during sustained swimming gradually increased the difference in MO_2_ between groups. At FT_max_, MO_2_ in the coronary-ligated group was ~ 38% lower than the sham-operated group (382 ± 37 and 616 ± 57 mg O_2_ h^−1^ kg^−1^, respectively; Fig. 2F), which was associated with a 43% lower cardiac output (62 ± 5 and 109 ± 8 mg O_2_ h^−1^ kg^−1^, respectively, Fig. 2G) because A-V O_2_ extraction was not different between groups (Fig. 2J). Again, the weaker cardiorespiratory performance of the coronary-ligated compared to the sham-operated group at FT_max_ was a direct result of heart rate being substantially lower (55 ± 7 and 110 ± 3 beats min^−1^; respectively, Fig. 2H), despite the significant increase in stroke volume (1.15 ± 0.17 and 0.90 ± 0.11 ml min^−1^; respectively, Fig. 2I). In fact, the heart rate at FT_max_ was significantly lower compared to routine values (64 ± 3 beats min^−1^) in the coronary-ligated group (*P* = 0.03). Thus, the primary mechanism for cardiorespiratory decline following coronary ligation was the inability to increase heart rate either with swimming or with acute warming. Moreover, the heartbeat was further compromised at FT_max_ in both groups, with all fatigued fish exhibiting bradycardia (as evident from visual inspection of individual recordings, data not shown), with or without irregular heartbeats (see Fig. S2).

Following recovery from fatigue, MO_2_, cardiac output and arterio-venous O_2_ extraction all recovered to routine baseline levels in both groups. While sham-operated fish retained a significantly elevated heart rate, coronary-ligated fish retained a significantly lower stroke volume relative to routine values.

There were general sex-specific differences in MO_2_, cardiac output and stroke volume across groups, whereby females had significantly lower values than males. Specifically, MO_2_ and arterio-venous O_2_ extraction were significantly lower at FT_max_ in females relative to males *P* = 0.014 and* P* = 0.005, respectively). Also, MO_2_, cardiac output, heart rate, stroke volume and arterio-venous O_2_ extraction were all numerically lower in coronary-ligated females at FT_max_ by 28% (123 mg O_2_ h^−1^ kg^−1^), 26% (20 ml min kg^−1^), 12% (8 beats min^−1^), 12% (0.15 ml min^−1^) and 28% (0.035 mg O_2_ ml^−1^), respectively. In the sham-operated group at FT_max_, MO_2_, cardiac output, stroke volume and A-V O_2_ extraction were all lower in females by 31% (225 mg O_2_ h^−1^ kg^−1^), 10% (12 ml min^−1^), 18% (0.20 ml min^−1^) and 26% (0.03 mg O_2_ ml^−1^), respectively, whereas heart rate was higher in females by 9% (10 beats min^−1^).

### Effects of coronary ligation, intense swimming and acute warming on blood oxygen carrying capacity and plasma variables

**Figure 3:**
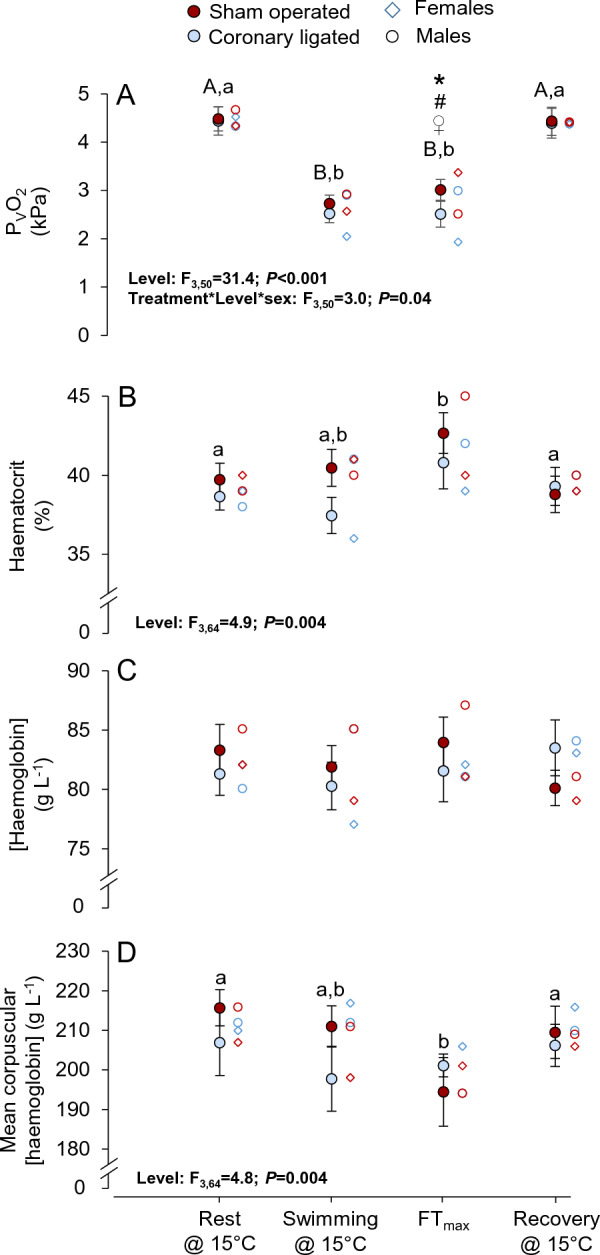
Effects of coronary ligation on venous oxygen supply and haematological variables in coho salmon (*Oncorhynchus kisutch*) during intense swimming and acute warming. The partial pressure of venous O_2_ (P_V_O_2_, **A**), haematocrit (**B**), [haemoglobin] (**C**) and the mean corpuscular [haemoglobin] in coronary-ligated (blue symbols) and sham-operated (red symbols) coho salmon at four experimental levels; routine conditions at 15 °C (0.3 body lengths s^−1^), during swimming (1.5 body lengths s^−1^) at 15 °C, at the temperature at which fish fatigued during an acute thermal challenge (i.e., the functional thermal maximum, FT_max_) and following recovery. Blue-lined (coronary-ligated) and red-lined (sham-operated) open circles depict male fish and diamonds depict female fish. The inset tables depict the outcome from the mixed models, which included the data obtained from each fish at the different levels. Only statistically significant (*P* ≤ 0.05) results are displayed here, see Tables S2 and S3 for full disclosure of sample sizes, variable values and the statistical results. In cases where significant interaction effects were detected between Treatment, Level and/or Sex, asterisks (*) and hash signs (#) denote statistically significant differences between treatment groups (sham-operated vs. coronary-ligated) and sexes (male vs. female), respectively, and ♀ signify differences in female fish between treatment groups (see result section for more details). For variables where a significant interaction between treatment group and experimental level (Treatment*Level) was detected (**A**), different capital and lower-case letters denote differences between the levels within sham-operated or coronary-ligated groups, respectively. For variables where no significant Treatment*Level interaction was detected (**B**–**D**), different single lower-case letters denote differences between the different experimental levels across treatment groups. Values are means ± SEM.

Routine P_V_O_2_ was statistically similar between coronary-ligated and sham-operated groups (4.4 ± 0.3 and 4.5 ± 0.3 kPa, respectively; Fig. 3A; see Tables S2 and S3 for values and statistics). P_V_O_2_ decreased with swimming at 15 °C, but remained similar between groups. However, at FT_max_, P_V_O_2_ was significantly lower in the coronary-ligated group compared to the sham-operated group (2.5 ± 0.3 and 3.0 ± 0.4 kPa, respectively; *P* = 0.047). The P_V_O_2_ during routine conditions and while swimming at 15 °C was slightly, but not significantly, lower in females relative to males across treatments, and a trend (*P* = 0.057) for the opposite was observed at FT_max_ (3.4 ± 0.3 and 2.6 ± 0.2 kPa, respectively; Table S2). In coronary-ligated fish, P_V_O_2_ only tended (*P* = 0.072) to be lower for females during swimming at 15 °C, and by FT_max_, P_V_O_2_ was significantly lower in females compared to males (2.0 ± 0.2 and 3.0 ± 0.2 kPa, respectively; *P* = 0.006). Moreover, the P_V_O_2_ at FT_max_ was significantly lower in female coronary-ligated fish relative to female sham-operated fish (2.0 vs. 3.4 kPa, respectively; *P* < 0.001).

Haematocrit, [haemoglobin] and mean corpuscular [haemoglobin] did not differ between groups during routine conditions, during swimming at 15 °C, at FT_max_ or following recovery (Fig. 3B–D, Table S2). Haematocrit was elevated above routine levels at FT_max_ in both groups but was restored to routine levels following recovery from fatigue. While [haemoglobin] remained unchanged across experimental levels, MCHC was significantly reduced at FT_max_ and was restored to routine levels following recovery.

**Figure 4:**
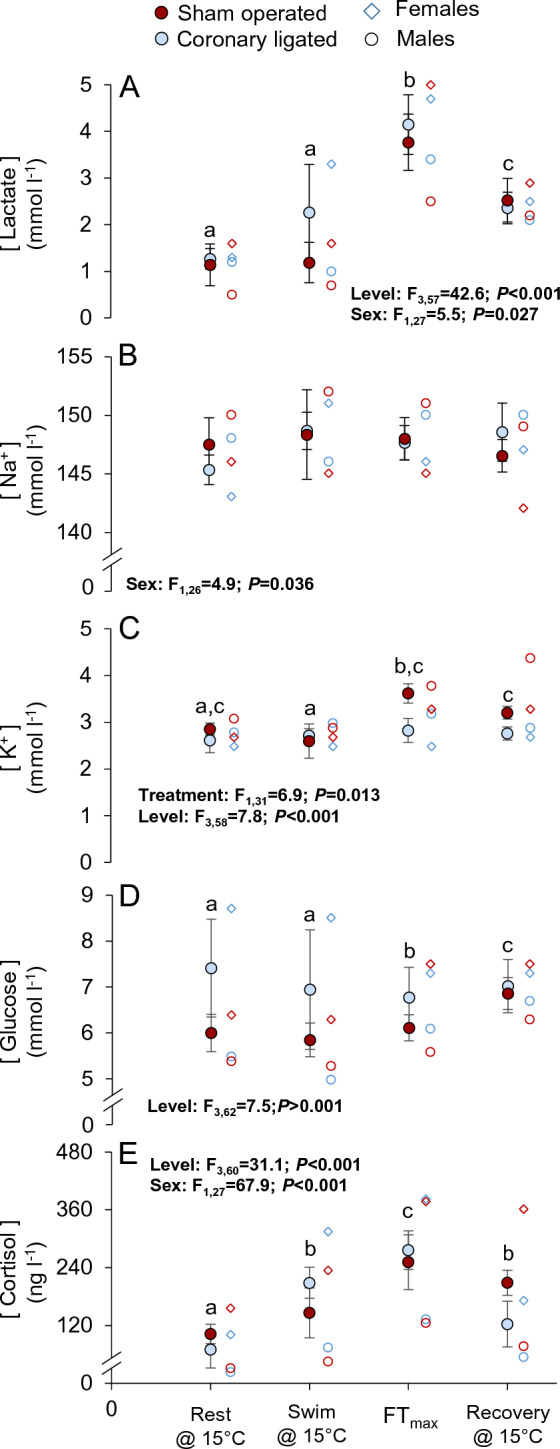
Effects of coronary ligation on plasma metabolite and hormone concentrations in coho salmon (*Oncorhynchus kisutch*) during intense swimming and acute warming. The partial pressure of plasma concentrations of lactate (**A**), Na^+^ (**B**), K^+^ (**C**), glucose (**D**) and cortisol (**E**) in coronary-ligated (blue symbols) and sham-operated (red symbols) coho salmon at four experimental levels; routine conditions at 15 °C (0.3 body lengths s^−1^), during swimming (1.5 body lengths s^−1^) at 15 °C, at the temperature at which fish fatigued during an acute thermal challenge (i.e., the functional thermal maximum, FT_max_) and following recovery. Blue-lined (coronary-ligated) and red-lined (sham-operated) open circles depict male fish and diamonds depict female fish. The inset tables depict the outcome from the mixed models, which included the data obtained from each fish at the different levels. Only statistically significant (*P* ≤ 0.05) results are displayed here, see Table S2 for sample sizes and variable values, and Table S4 for full disclosure of the statistical results. Different lower-case letters denote differences between the different experimental levels across treatment groups. Values are means ± SEM.

Plasma [Lactate], [Na^+^], [K^+^], [Glucose] or [Cortisol] did not differ between the two treatment groups (Fig. 4A–E, see Tables S2 and S4 for values and statistics). Plasma [Lactate] increased with warming in both groups and was significantly elevated at FT_max_, and while it decreased following recovery from FT_max_, it remained higher relative to routine values (Fig. 4A). Plasma [Lactate] was generally higher in females (Table S2). Plasma [Na^+^] did not change across experimental levels in either group (Fig. 4B), but there was a general effect of lower [Na^+^] in females across treatments. Plasma [K^+^] was significantly elevated at FT_max_ in both groups (Fig. 4C), and the significant treatment effect seemingly reflected a lower [K^+^] in the coronary-ligated group at FT_max_. Plasma [Glucose] increased across levels in the sham-operated group, however, an opposite response was observed in the coronary-ligated group (Fig. 4D). Plasma [Glucose] and [Cortisol] were in general higher in females. Plasma [Cortisol] increased across levels at FT_max_ and decreased following recovery from fatigue but remained elevated above routine levels (Fig. 4E).

### Tissue metabolite concentration and enzyme activities

There were no differences between coronary-ligated and sham-operated groups for [Lactate] in both cardiac (6.6 ± 0.9 vs. 9.9 ± 1.9 mmol kg^−1^, respectively) and white muscle (24.3 ± 4.6 vs. 18.3 ± 2.3 mmol kg^−1^, respectively), nor were there any difference between sexes.

**Figure 5:**
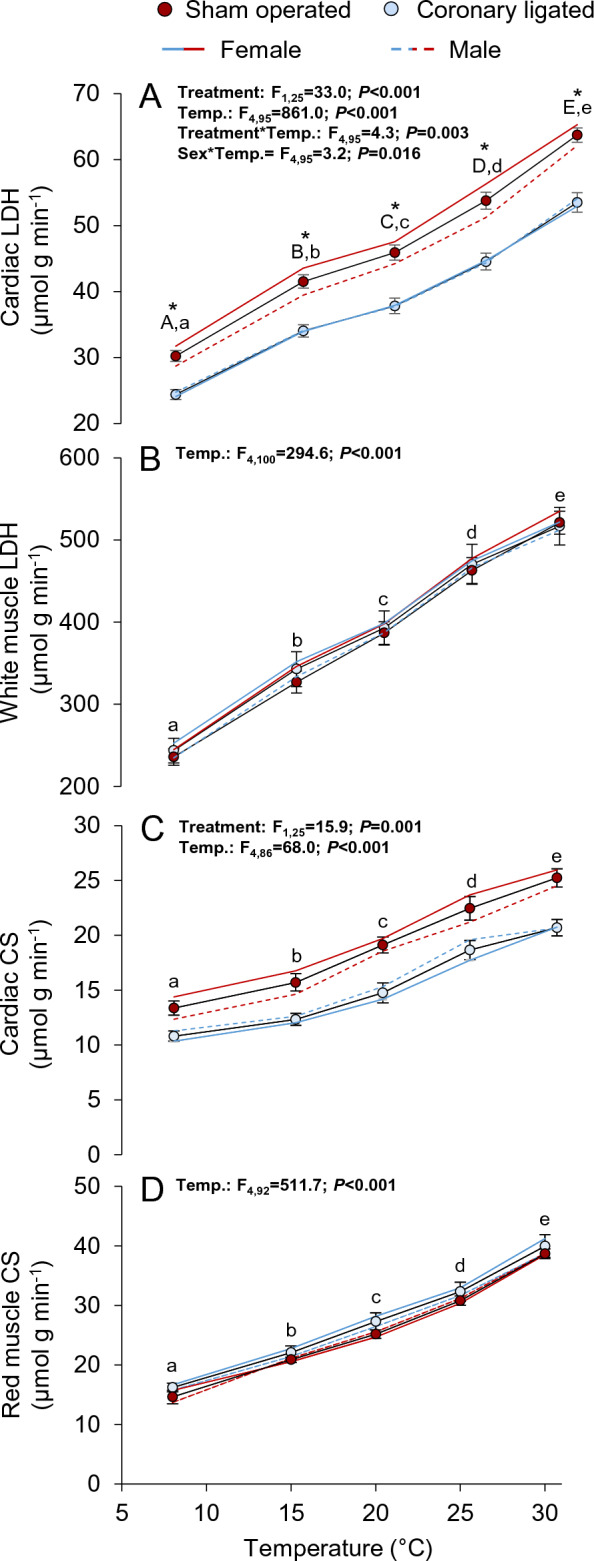
Effects of coronary ligation and warming on aerobic and anaerobic enzymatic activities of cardiac ventricular and muscle tissues from coho salmon (*Oncorhynchus kisutch*). Enzymatic activities and thermal sensitivity of lactate dehydrogenase (LDH) from cardiac (**A**) and white muscle tissues (**B**), and the activities of Citrate synthase (CS) from cardiac tissues (**C**) and white muscle tissues (**D**). The data for coronary-ligated or sham-operated female (blue or red solid lines, respectively) and male (blue and red dashed lines, respectively) fish are depicted in each panel (see Table S5 for sample sizes across groups and temperatures). The inset tables depict the outcome from mixed models, which included the data obtained at 8, 15, 20, 25 and 30 °C. Only statistically significant (*P* ≤ 0.05) results are displayed here, see Table S6 for full disclosure of the statistical results. Asterisks (*) denote statistically significant differences between treatment groups (sham-operated vs. coronary-ligated). In (**A**), where a significant interaction between treatment group and experimental temperature (Treatment*Temperature) was detected, different capital and lower-case letters denote differences between the different temperatures within sham-operated or coronary-ligated groups, respectively. For variables where no significant Treatment*Temperature interaction was detected (**A**–**D**), different single lower-case letters denote differences between the different temperatures across treatment groups. Values are means ± SEM.

The activity of LDH in cardiac muscle was 16–19% lower in coronary-ligated relative to sham-operated fish across temperatures, and LDH activity increased significantly with warming in both groups (Fig. 5A, see Table S5 for statistics). In the sham-operated group, a significant interaction was found between sex and temperature, which was attributed to a lower cardiac LDH activity at 25 °C in females. However, there were no sex-specific differences at the other temperatures. White muscle LDH activity did not differ between groups and increased significantly with warming (Fig. 5B).

CS activity in both cardiac and red muscle increased significantly with temperature (Fig. 5C,D). While red muscle CS activity was similar in both groups (Fig. 5D), the coronary-ligated group displayed a 18–23% lower cardiac CS activity compared to the sham-operated group across temperatures (Fig. 5C).

## Discussion

We investigated the importance of coronary circulation for the cardiorespiratory performance of coho salmon during sustained swimming at 60% of their U_crit_ whilst being acutely warmed to temperatures above T_opt_ for metabolic performance in this population (~ 14 °C^30^). Coronary ligation stopped the flow of oxygenated blood to the outer ~ 28% of the ventricle (the compact myocardium). Our findings clearly demonstrate that coronary perfusion is of great importance for governing warming tolerance during swimming in this species, but of lesser importance for routine performance at temperatures close to T_opt_. Coronary ligation reduced FT_max_ by 4.4 °C relative to fish with an intact coronary supply, and some coronary-ligated fish fatigued at 15 °C which just exceeds T_opt_. These findings reinforce previous observations highlighting the importance of the coronary circulation for upper thermal tolerance (coronary ligation reduces CT_max_ by 1–1.5 °C in resting rainbow trout^21,24,25^) and swimming performance in salmonids (coronary ligation reduced U_crit_ by 35% in chinook salmon,^27^). Moreover, the novel experimental approach of combining acute warming with sustained swimming, which more closely reflects the fluctuating river conditions of migrating salmon, revealed not only the drastic decline in FT_max_ but also the cardiorespiratory changes associated with swimming fatigue at this supra-optimal temperature. The coronary circulation clearly has a crucial role for the capacity of salmon to sustain intense swimming when faced with extreme warming. Such conditions may be encountered on a daily basis^36^ or throughout the duration of their spawning migration^37^, and during extreme heat events which are predicted to become more frequent and severe with exacerbated climate warming^38^.

Our results provide a clear mechanistic linkage between the deterioration of cardiorespiratory performances and FT_max_ in coho salmon lacking coronary blood flow to the ventricle. Furthermore, we confirmed indirectly the importance of previously reported increases in coronary blood flow during swimming and with acute warming^20,21^, conditions that elevate whole animal metabolic demand. For example, coronary ligation reduced cardiac output and MO_2_ during sustained swimming near T_opt_ (1.5 bl s^−1^ at 15 °C) mainly due to an abrupt and drastic decline in heart rate. This bradycardia then persisted throughout the warming protocol. Normally, cardiac output in salmonids increases during swimming by increasing both heart rate and stroke volume, as observed here in sham-operated fish and in previous works^9,10,39,40^, and during acute warming by elevating only heart rate, as observed by Steinhausen and colleagues^10^, who performed a similar experimental protocol on sockeye salmon (swim speed: 1.35 bl s^−1^, 75% of U_crit_; heating rate: 2 °C h^−1^, but without manipulation of the coronary artery). Tissue oxygen delivery is further aided by the increase in arterio-venous O_2_ extraction, which decreases P_V_O_2_ as observed here in sham-operated coho salmon. However, immediately before FT_max_ (average temperature: 21.3 ± 0.4 °C), the sham-operated group had reached a heart rate of ~ 115 beats min^−1^, i.e., close to their upper limit of ~ 120 beats min^−1^^41^ (see also^9,10,24,25,40^), while the heart rate of the coronary-ligated group was only 57 beats min^−1^ (average temperature: 17.1 ± 1.1 °C), which was even lower than routine heart rate. Furthermore, a compensatory increase in stroke volume for this bradycardia was not possible beyond the increase associated with swimming. Given that Steffensen and Farrell^19^ demonstrated that coronary ligation reduced ventral aortic blood pressure of rainbow trout swimming at 1.5 bl s^−1^ at 15 °C, we suggest coronary ligation impaired cardiac contractility and thus stroke volume during the combined stress of swimming and warming. Moreover, in resting rainbow trout, coronary ligation reduced routine stroke volume and cardiac output during warming alone^24,25^. Consequently, the severe incapacity to elevate heart rate reduced cardiac output and MO_2_ in coronary-ligated fish (by 50, 43 and 38%, respectively) and presumably caused a severe mismatch between tissue oxygen supply and demand of swimming muscles, causing fatigue at lower temperatures. This mechanistic interpretation is consistent with our correlational analysis that showed heart rate explaining 75% of the variation in FT_max_ (see Supplementary Material S1); those individuals with coronary ligation that better maintained an elevated heart rate, cardiac output and MO_2_ (explaining 55 and 50% of the variation, respectively) fatigued at higher temperatures. This conclusion is similar to that for sockeye salmon^10^, in which swimming performance was restricted by an inability to elevate cardiac output and MO_2_ at high temperatures. Yet, in sockeye salmon^10^, there were no significant relationships between cardiorespiratory performances and FT_max_, which is what we found for our sham group.

A compensatory improvement of arterial oxygen carrying capacity at higher temperatures was not seen in either group. There was no change in [haemoglobin] and the modest elevation in hematocrit at FT_max_ in both groups was associated with a decrease in corpuscular [haemoglobin], which is indicative of erythrocyte swelling as opposed to a splenic release of erythrocytes, which can occur during warming and swimming^42–45^. Nor was there any elevation in arterio-venous O_2_ extraction which could have augmented MO_2_ at higher temperatures in coronary-ligated fish, as seen previously in swimming rainbow trout^46^, and in resting salmonids exposed to acute warming^12,47^. Collectively, the current findings clearly demonstrate that perfusion of the compact myocardium is crucial for properly elevating cardiorespiratory function to support swimming performance in salmonids during warming.

We cannot fully elucidate the cardiophysiological underpinnings of the bradycardia observed in coronary-ligated fish while swimming at 15 °C, and close to FT_max_ in both treatment groups in the current experiment. Notably, the P_V_O_2_ and thus the driving pressure for luminal myocardial oxygen diffusion diminished when swimming started. Perhaps a P_V_O_2_ threshold (i.e., ~ 2.5 kPa) was approached at the outset of swimming which limited cardiac oxygen supply and constrained cardiac energy production and function, as proposed by Davie and Farrell^41^. Furthermore, at FT_max_, the P_V_O_2_ for sham-operated and coronary-ligated fish (3.0 and 2.5 kPa, respectively) are within the P_V_O_2_ threshold range at which cold (6–10 °C) and warm (13–16 °C) acclimated rainbow trout reached their U_crit_^18^. Thus, both groups may have reached a P_V_O_2_ threshold that constrained cardiac function at FT_max_. Cardiac dysrhythmias similar to those seen here were reported in 57% of sockeye salmon prior to fatigue (U_crit_) at high temperatures^9^. The mechanistic link between ventricular oxygen supply and bradycardia/dysrhythmia requires future study.

Another possible driver of bradycardia near FT_max_ is an extracellular metabolic acidosis associated with the observed high plasma [lactate]; acidosis impairs cardiac contractility in fish^48,49^, which could also have constrained cardiac contraction force generation and the capacity to increase stroke volume and cardiac output during the bradycardia. Furthermore, the positive correlation between FT_max_ and cardiac LDH activity at FT_max_ for the coronary-ligated group suggests that individuals with either higher cardiac anaerobic ATP production, or better ability to metabolize lactate to ATP, faired better at higher temperatures (Fig. S4). The positive relationship between FT_max_ and cardiac CS activity at FT_max_ also suggest that coronary-ligated fish with higher cardiac aerobic capacity endured to higher temperatures. We believe the lower activities of cardiac LDH and CS in the ligated group reflect the loss of viable myocardium, which had been rendered hypoxic or anoxic for > 16 h at the time of tissue harvesting. Nevertheless, compensatory changes are possible following the ligation procedure because 28–52 days after coronary ligation, rainbow trout increased CS activity in the spongy myocardium by 27% compared to sham-operated fish, which resulted from a 18% net increase in ventricular CS activity alongside a 9% decrease in CS activity in compact myocardium^50^. We saw no equivalent short-term compensation here.

Future experiments should focus on a potential impairment of electrical conduction in the ventricular myocardium; Electrocardiogram (ECG) recordings have shown that a similar type of bradycardia, as observed here, occurred in rainbow trout following an exhaustive chase protocol to elicit maximum cardiorespiratory performance^51^, which was attributed to second degree atrio-ventricular block^52,53^. The atrio-ventricular canal of fish conducts action potentials from the sinoatrial pacemaker region to the ventricle, and in salmonids contains compact myocardium perfused by the coronaries^54^. The atrio-ventricular conduction rates could therefore be particularly sensitive to coronary ligation (and arteriosclerosis) and could explain the bradycardia observed in ligated fish swimming at 15 °C. Indeed, the compact myocardium remains viable (but substantially degraded) following acute coronary ligation in rainbow trout^55^, indicating that the compact myocardium can to some extent extract oxygen from the luminal supply. Normally, peak coronary blood flow in salmonids occurs during cardiac relaxation between heartbeats (diastole)^56,57^, meaning that the higher heart rates during swimming and warming, which shorten these diastolic periods, likely decrease coronary blood flow to the compact myocardium and the atrio-ventricular canal. Thus, insufficient coronary blood flow may trigger the irregular heartbeats and resulting bradycardia observed in swimming sham-operated coho salmon close to FT_max_ and their upper limit for heart rate. Potential oxygen deprivation of the atrio-ventricular canal in fish is, to our knowledge, unexplored and deserves further scrutiny.

We anticipated greater negative impacts of coronary ligation on cardiorespiratory performance in females, which would lower FT_max_ relative to males. Our prediction was based on sexually maturing female rainbow trout exhibiting a higher resting coronary blood flow compared to males, which lowered their scope for increasing coronary blood flow during acute warming to 18 °C (~ 8° below CT_max_ in this strain)^21^. A “greedy gonads hypothesis” was advanced for these observations: increased O_2_ extraction by the maturing gonads in females is hypothesized to reduce P_V_O_2_ more than males (but see^58^) and thereby increased the heart’s reliance on the coronary oxygen supply during warming. The current data lend partial support for this idea despite low sample sizes for the sex-specific comparisons. Notably, P_V_O_2_ in swimming coronary-ligated females was numerically lower relative to males and became significantly so at FT_max_. Since this situation was not as extreme in sham-operated females, it is possible that the impairment of cardiorespiratory capacity following coronary ligation resulted in a greater mismatch between oxygen delivery and demand of the tissues including the maturing gonads, which lowered P_V_O_2_ in female salmon at FT_max_. A lower P_V_O_2_ may in turn have further exacerbated the deterioration of cardiac capacities, and may explain the relatively greater reductions in cardiac output, heart rate and stroke volume at FT_max_ in coronary-ligated females compared to sham-operated females, relative to male fish within treatment groups.

Our data at FT_max_, demonstrating lower cardiac stroke volume, cardiac output and MO_2_ in females has similarities with previous findings in pink salmon (*Oncorhynchus gorbuscha*), in which females swimming maximally at temperatures ranging between 8 and 28 °C displayed lower maximum cardiac output, metabolic rate and aerobic scope relative to males^59^. These observations most likely reflect the fact that maturing females have a smaller ventricle, as observed here and in other Pacific salmon species^59,60^, which could limit increases in cardiac stroke volume and cardiac output^61^. Surprisingly, however, and in contrast to our prediction, the general reduction in cardiorespiratory capacities in females did not negatively impact FT_max_ relative to males. It is possible that females relied to a greater extent on anaerobic metabolism to sustain energy production and swimming performance at higher temperatures, as indicated here by the higher plasma [glucose] and [lactate] at FT_max_ in females. This would support several field-based telemetry studies showing that migrating female sockeye salmon rely more on anaerobic swimming than aerobic swimming^7,62–64^. Nevertheless, in the context of fish migration, a greater reliance on anaerobic metabolism likely comes with negative temporal side-effects, as discussed by Hinch and colleagues^7^. For example, increased anaerobic metabolism and lactate accumulation would lengthen post-exercise recovery time which could result in prolonged migration, increased susceptibility to predation, and elevated mortality in female salmon. The sex-specific differences in cardiorespiratory performances reported here, and in mortality during spawning migration as summarized by Hinch et al.^7^, warrant further study with a continued emphasis on the relevance of the coronary circulation for performances relating to migratory capacity in salmonids.

In conclusion, we show that coronary perfusion is crucial for determining not only the cardiorespiratory capacity but also acute warming tolerance in coho salmon during sustained swimming. Thus, it is rational to expect that coronary arteriosclerosis would impair the progress of salmon on their essential, upriver spawning migrations, and that these impairments would become more relevant with climate warming. The current data also provides evidence that the influence of the coronary circulation on cardiorespiratory performance in salmon can be sex-specific, and that the cardiorespiratory capacity is in general lower in females.

### Supplementary Information

Supplementary Information.

## Data Availability

The original data relating to this manuscript is available as electronic supplementary material (10.6084/m9.figshare.24599298).
