# Supplementary material for: Impairing cardiac oxygen supply in swimming coho salmon compromises their heart function and tolerance to acute warming
Source: Sci Rep. 2023 Dec 1;13:21204. doi: 10.1038/s41598-023-47713-5 (PMC10692232; doi:10.1038/s41598-023-47713-5)
Supplement: Supplementary file 1 — Supplementary Information. [file 41598_2023_47713_MOESM1_ESM.docx]

Supplementary material for Ekström et al.,”Impairing cardiac oxygen supply in swimming coho salmon compromises their heart function and tolerance to acute warming”


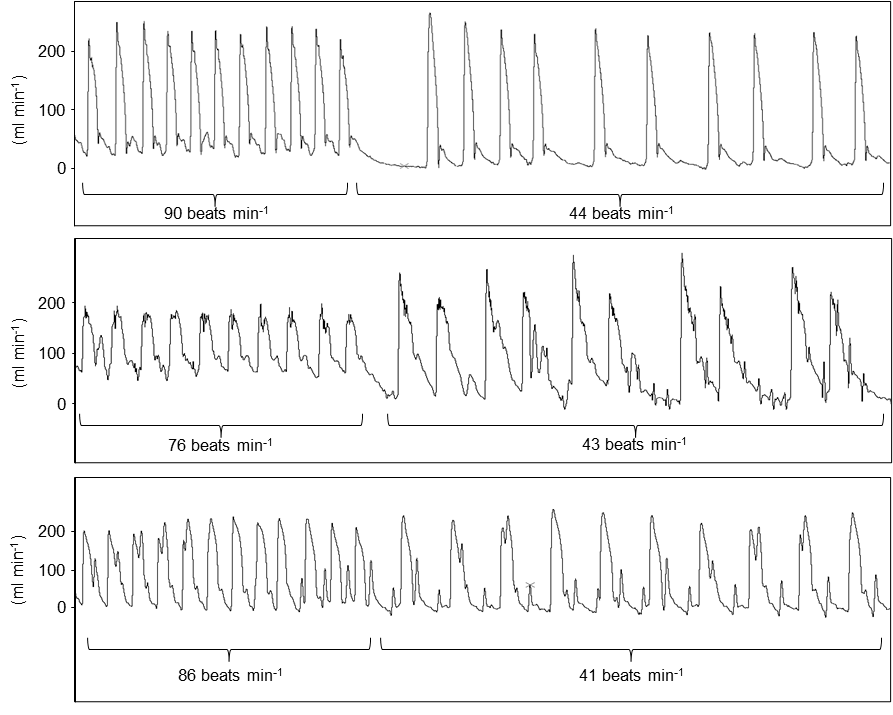


**Figure S1. Onset of bradycardia in swimming coronary ligated coho salmon (*Oncorhynchus kisutsch*).** Three examples of the sudden and drastic decline in heart rate (bradycardia) that occurred in 11 out of 14 coronary ligated fish shortly after the onset of swimming at 1.5 bl s^-1^ at 15°C. Heart rate was determined by counting the pulsatile cardiac contraction cycles from the raw cardiac output traces depicted in each panel. The numbers in each panel depict the calculated average heart rates for the periods within brackets.


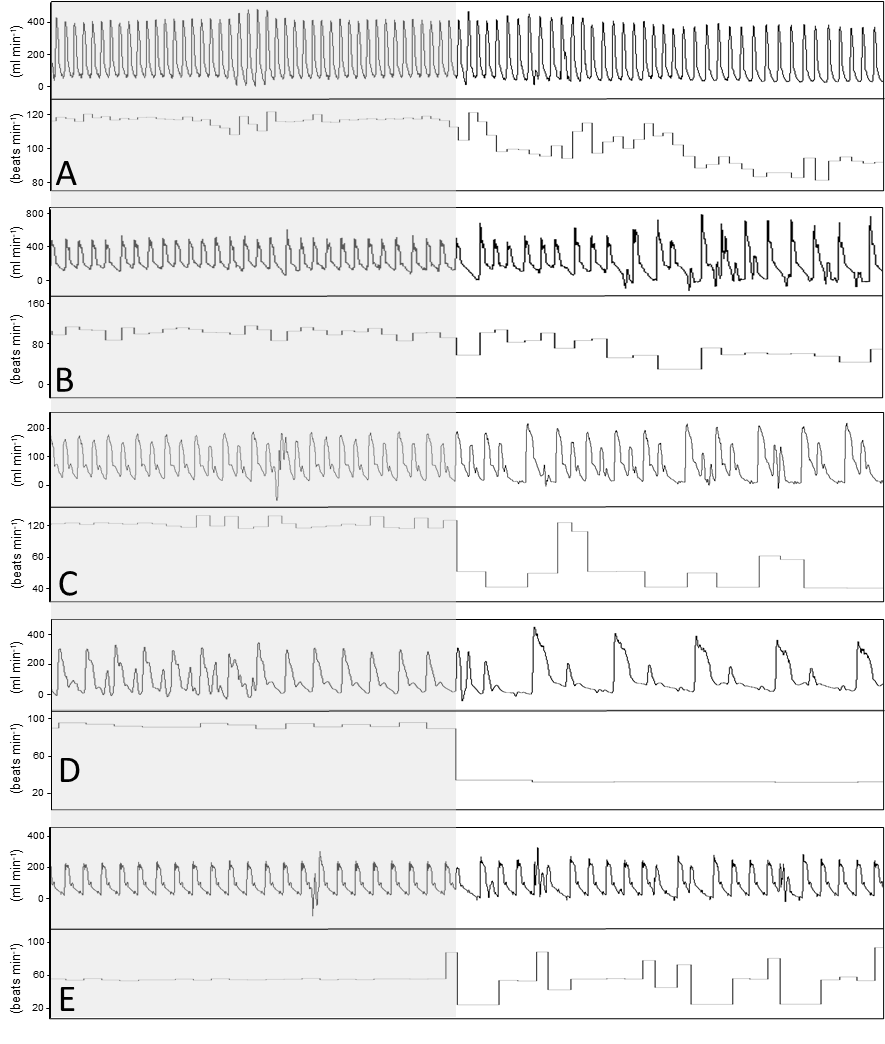


**Figure S2. Onset of bradycardia at which fish fatigued in swimming sham operated and coronary ligated coho salmon (*Oncorhynchus kisutsch*).** Five examples from sham operated (A,B) or coronary ligated (C-E) individual fish of the decline in heart rate that occurred around the point at which the fish fatigued and stopped swimming (*i.e*., the functional thermal maximum, FT_max_). Heart rate was determined by counting the pulsatile cardiac contraction cycles from the raw cardiac output traces (top section within each panel). The shaded area depicts the periods preceding the bradycardia, which was caused by a progressive lowering of cardiac contraction frequency or by what appeared to be arrhythmic heartbeats.

*Correlations between FT_max_, cardiorespiratory performances, haematological and blood plasma variables and muscle enzyme activities*

The relationship between FT_max_ and a failed cardiorespiratory capacity was more prominent in the coronary-ligated group, as demonstrated by statistically significant positive correlations between FT_max_ and cardiac output (P_corr_=0.55; *P*=0.041, Fig. S3) and heart rate (P_corr_=0.74; *P*=0.002), and a trend for a negative correlation between FT_max_ and stroke volume (P_corr_=-0.41; *P*=0.14). There was also a trend for a positive correlation between FT_max_ an MO_2_ (P_corr_=0.50; *P*=0.066). No such relationships were observed in the sham-operated group. Moreover, there were positive correlations between FT_max_ and [Haemoglobin] (P_corr_=0.59; P=0.03; Fig. S4) in the coronary-ligated group, and between FT_max_ and haematocrit (P_corr_=0.67; P=0.01) in the sham-operated group. FT_max_ was positively correlated with cardiac LDH activity (P_corr_=0.57; *P*=0.032, Fig. S4) and cardiac CS activity (P_corr_=0.67; *P*=0.018) in the coronary-ligated group. A trend towards a positive relationship between red muscle CS activity and FT_max_ was observed in the coronary-ligated group (P_corr_=0.52; *P*=0.057).

There were statistical trends for positive correlations between P_V_O_2_ and cardiac output (P_corr_=0.54; *P*=0.07; Fig. S5) and MO_2_ (P_corr_=0.55; *P*=0.06) in the coronary-ligated but not the sham-operated group. Moreover, there was a negative correlation between P_V_O_2_ and plasma [Lactate] (P_corr_= -0.75; *P*=0.005), while a positive relationship was found between P_V_O_2_ and [K^+^] (P_corr_=0.62; *P*=0.031). In the coronary-ligated group, plasma [Lactate] was negatively correlated with cardiac output (P_corr_=-0.53; *P*=0.05, Fig. S6) and MO_2_ (P_corr_=-0.64; *P*=0.014), and plasma [K^+^] was positively correlated with cardiac output (P_corr_=0.77; *P*=0.001). Plasma [Lactate] was negatively correlated with stroke volume (P_corr_=0.63; *P*=0.022) in the sham-operated group. Cardiac LDH was negatively correlated with stroke volume (P_corr_=-0.66; *P*=0.01) in the coronary-ligated group, and there was also a statistical trend towards a positive correlation between for cardiac LDH activity and heart rate (P_corr_=0.52; *P*=0.055). Cardiac LDH activity was positively correlated with cardiac output (P_corr_=0.49; *P*=0.087), stroke volume (P_corr_=0.54; *P*=0.058) and MO_2_ (P_corr_=0.56; *P*=0.060) in the sham-operated group.

**
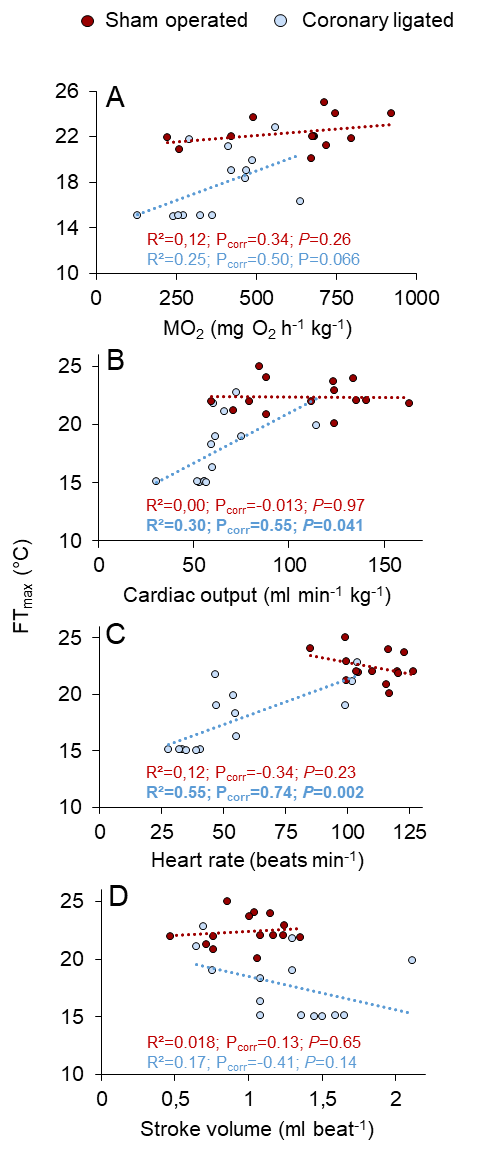
**

**Figure S3. Correlations between the functional thermal maximum (FT_max_) and cardiorespiratory performances at FT_max_ in coronary ligated and sham operated coho salmon (*Oncorhynchus kisutsch*).** The Pearson correlation coefficient (P_corr_) signifies the strength of the linear relationship between the variables and the *P* values show whether the correlations were statistically significant within treatment groups (*P* ≤ 0.05).


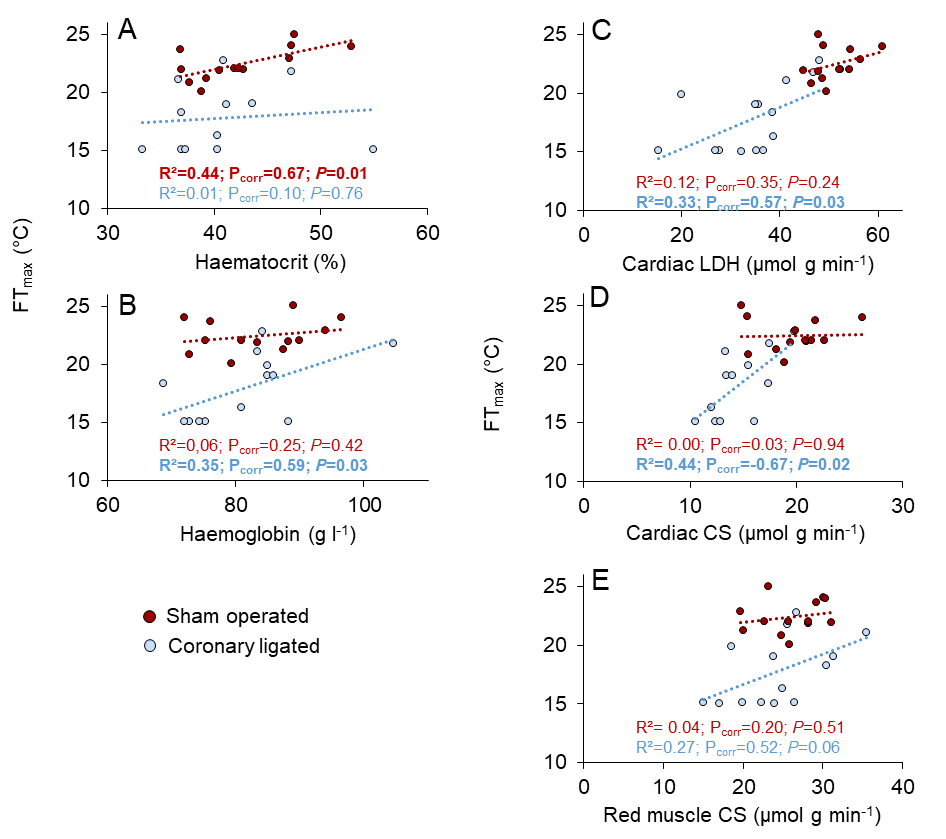


**Figure S4. Correlations between the functional thermal maximum (FT_max_), haematological blood oxygen transport capacity and enzymatic activities of Lactate dehydrogenase (LDH) and Citrate synthase (CS) at FT_max_ in coronary ligated and sham operated coho salmon (*Oncorhynchus kisutsch*).** The Pearson correlation coefficient (P_corr_) signifies the strength of the linear relationship between the variables and the *P* values show whether the correlations were statistically significant within treatment groups (*P* ≤ 0.05).

**
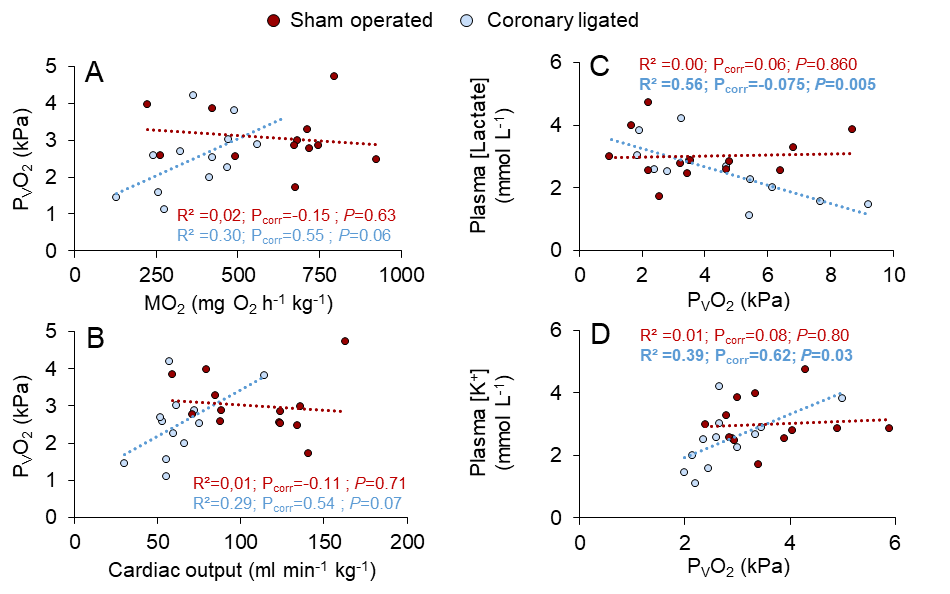
**

**Figure S5. Correlations between cardiac oxygen availability (the partial pressure of venous oxygen, P_V_O_2_), cardiorespiratory performance and blood plasma metabolites at the functional thermal maximum (FT_max_) in coronary-ligated and sham-operated coho salmon (*Oncorhynchus kisutsch*).** The Pearson correlation coefficient (P_corr_) signifies the strength of the linear relationship between the variables. The *P* values show whether the correlations were statistically significant within treatment groups (*P* ≤ 0.05).


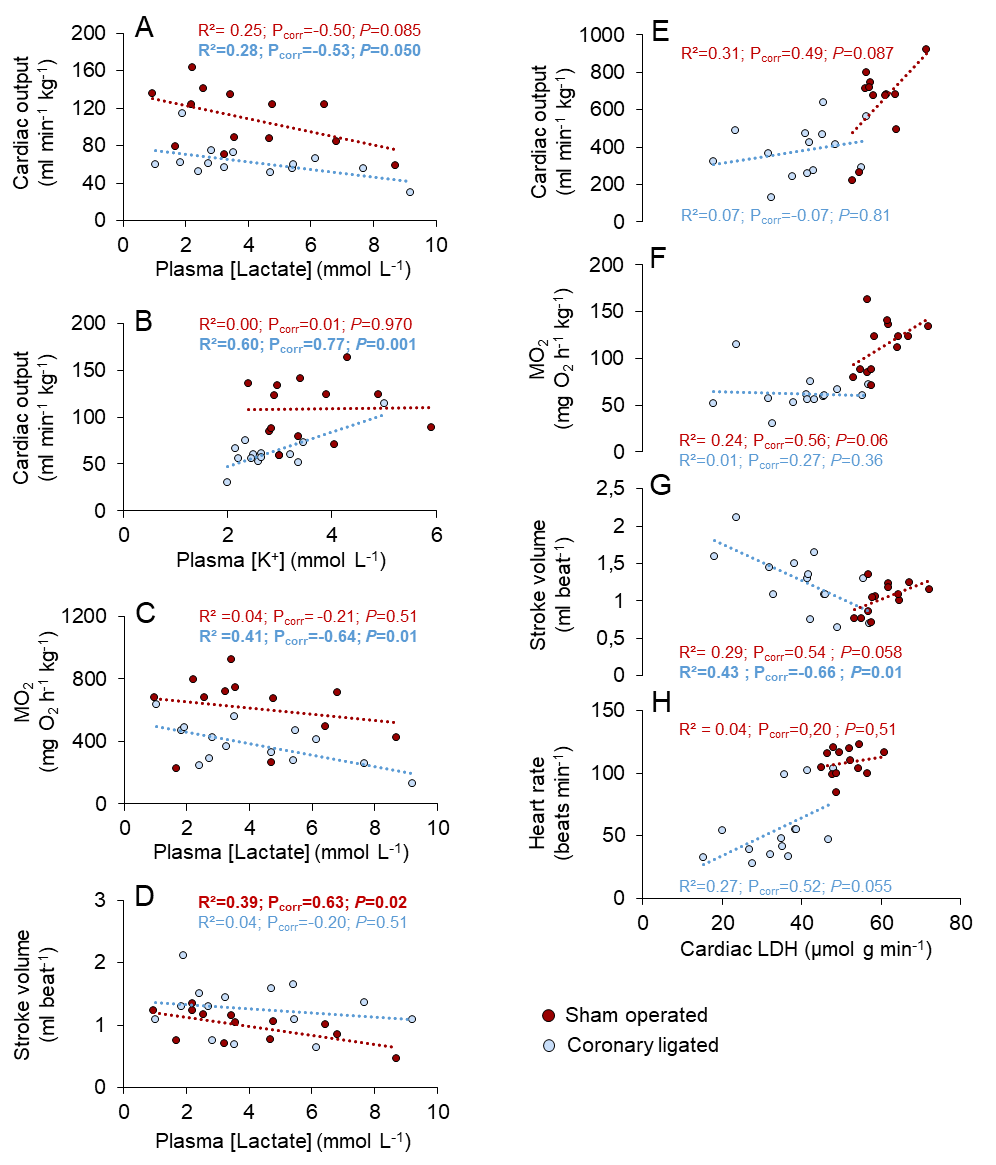


**Figure S6. Correlations between cardiorespiratory performances, plasma metabolite concentrations and enzymatic activities of Lactate dehydrogenase (LDH) and Citrate synthase (CS) at the functional thermal maximum (FT_max_) in coronary-ligated and sham-operated coho salmon (*Oncorhynchus kisutsch*).** The Pearson correlation coefficient (P_corr_) signifies the strength of the linear relationship between the variables. The *P* values show whether the correlations were statistically significant within treatment groups (*P* ≤ 0.05).

| Table S1. Statistical details from the mixed model analyses performed on the data illustrated in figure 2. | | | | | |
| --- | --- | --- | --- | --- | --- |
|  | MO_2_ | Cardiac output | Heart rate | Stroke volume | A-V O_2_ extraction |
| Level | ***F*_3,69_=121.6; *P*>0.001** | ***F*_3,69_=83.1; *P*>0.001** | ***F_1,41_*=4.8; *P*>0.01** | ***F*_3,69_=54.2; *P*>0.001** | ***F_3,71_*=47.3;** ***P*>0.001** |
| Treatment | ***F_1_*_,44_=22.1; *P*>0.001** | ***F*_1,33_=30.2; *P*>0.001** | ***F_1,41_*=28.7;** ***P*>0.001** | *F*_1,34_=1.1; *P=*0.30 | *F_1,42_*=0.1; *P*=0.77 |
| Sex | ***F_1_*_,44_=5.4; *P=*0.024** | ***F*_1,33_=7.1; *P=*0.012** | *F_3,73_*=0.0; *P=*0.995 | ***F*_1,34_ =8.6; *P*>0.01** | *F_1,42_*=0.2; *P*=0.63 |
| Level*Treatment | *F*_3,69_=1.4; *P*=0.25 | ***F*_3,69_=8.2;** ***P*>0.001** | ***F_3,73_*=23.5;** ***P*>0.001** | ***F*_3,69_=10.4; *P*>0.001** | *F_3,71_*=0.3; *P*=0.82 |
| Level*Sex | ***F*_3,69_=3.0; *P=*0.035** | *F*_3,69_=0.9; *P*=0.41 | *F_3,73_*=0.8; *P*=0.53 | *F*_3,69_=2.4; *P*=0.08 | ***F_3,71_*=3.3; *P=*0.02** |
| Treatment*Sex | *F_1_*_,44_=1.0; *P*=0.32 | *F*_1,33_=33.1; *P*=0.80 | *F_1,41_*=1.7; *P*=0.20 | *F*_1,34_=0.6; *P*=0.44 | *F_1,42_*=0.0; *P*=0.94 |
| Level*Treatment*Sex | *F*_3,69_=0.17; *P*=0.92 | *F*_3,69_=0.5; *P*=0.69 | *F_3,73_*=0.3; *P*=0.80 | *F*_3,69_=0.0; *P*=0.99 | *F_3,71_*=0.1; *P*=0.96 |
| *Statistically significant (P<0.05) model outcomes are depicted in bold. MO_2_ = Oxygen consumption rate* | | | | | |

| Table S2. Values and sample sizes for the hematological and blood plasma variables illustrated in Figures 3 and 4 in coronary ligated and sham operated coho salmon (*O.kisutsch*). | | | | | | | | | | | | | | | | |
| --- | --- | --- | --- | --- | --- | --- | --- | --- | --- | --- | --- | --- | --- | --- | --- | --- |
|  | Rest at 15°C | | | | Swimming at 15°C | | | | Fatigue (FT_max_) | | | | Recovery | | | |
|  | Sham operated | | Coronary ligated | | Sham operated | | Coronary ligated | | Sham operated | | Coronary ligated | | Sham operated | | Coronary ligated | |
|  | F | M | F | M | F | M | F | M | F | M | F | M | F | M | F | M |
| P_V_O_2_ (kPa) | 4.5±0.3 | | 4.4±0.3 | | 2.7±0.2 | | 2.5±0.2 | | 3.0±0.2 | | 2.5±0.3 | | 4.4±0.3 | | 4.4±0.3 | |
|  | 4.4±0.4 | 4.7±0.3 | 4.5±0.6 | 4.3±0.2 | 2.6±0.2 | 3.0±0.2 | 2.1±0.1 | 2.9±0.2 | 3.4±0.3 | 2.6±0.2 | 2.0±0.2 | 3.0±0.4 | 4.4±0.4 | 4.4±0.4 | 4.4±0.5 | 4.4±0.5 |
| *N, treatment groups* | *13* | | *14* | | *12* | | *8* | | *13* | | *12* | | *13* | | *14* | |
| *N, sexes* | *8* | *5* | *7* | *7* | *8* | *4* | *4* | *4* | *7* | *6* | *6* | *6* | *7* | *6* | *7* | *7* |
| Haematocrit (%) | 40±1 | | 39±1 | | 40±1 | | 38±1 | | 43±1 | | 41±2 | | 39±1 | | 39±1 | |
|  | 40±2 | 39±1 | 39±1 | 38±1 | 41±2 | 40±1 | 36±1 | 41±1 | 40±2 | 45±3 | 39±2 | 42±3 | 39±1 | 40±2 | 39±1 | 40±2 |
| *N, treatment groups* | *14* | | *15* | | *15* | | *10* | | *14* | | *12* | | *14* | | *14* | |
| *N, sexes* | *8* | *6* | *8* | *7* | *8* | *7* | *6* | *4* | *7* | *7* | *6* | *6* | *7* | *7* | *7* | *7* |
| [Haemoglobin] (g l^-1^) | 83±2 | | 81±2 | | 82±2 | | 80±2 | | 84±2 | | 82±3 | | 80±1 | | 83±2 | |
|  | 82±3 | 85±3 | 82±3 | 80±2 | 79±2 | 85±2 | 77±3 | 85±2± | 81±2 | 87±3 | 82±2 | 81±5± | 79±1 | 81±3 | 83±2 | 84±4 |
| *N, treatment groups* | *14* | | *15* | | *15* | | *10* | | *14* | | *12* | | *14* | | *14* | |
| *N, sexes* | *8* | *6* | *8* | *7* | *8* | *7* | *6* | *4* | *7* | *7* | *6* | *6* | *7* | *7* | *7* | *7* |
| MCHC | 211±5 | | 211±3 | | 204±5 | | 215±5 | | 198±4 | | 200±5 | | 208±4 | | 213±3 | |
|  | 207±8 | 216±5 | 210±3 | 212±5 | 198±8 | 211±5 | 217±7 | 212±10 | 201±3 | 194±9 | 206±8 | 194±7 | 206±5 | 209±7 | 216±5 | 210±4 |
| *N, treatment groups* | *14* | | *15* | | *15* | | *10* | | *14* | | *12* | | *14* | | *14* | |
| *N, sexes* | *8* | *6* | *8* | *7* | *8* | *7* | *6* | *4* | *7* | *7* | *6* | *6* | *7* | *7* | *7* | *7* |
| Lactate (mmol l^-1^) | 1.1±0.4 | | 1.3±0.2 | | 1.1±0.4 | | 2.3±1.0 | | 3.8±0.6 | | 4.2±0.6 | | 2.5±0.5 | | 2.4±0.3 | |
|  | 1.6±0.8 | 0.5±0.1 | 1.3±0.1 | 1.2±0.5 | 1.6±0.8 | 0.7±0.1 | 3.3±1.8 | 1.0±0.2 | 5.0±1.0 | 2.5±0.4 | 4.7±0.8 | 3.4±1.0 | 2.9±0.3 | 2.2±0.8 | 2.5±0.5 | 2.1±0.5 |
| *N, treatment groups* | *14* | | *15* | | *15* | | *9* | | *14* | | *14* | | *13* | | *14* | |
| *N, sexes* | *8* | *6* | *9* | *6* | *8* | *7* | *5* | *4* | *7* | *7* | *8* | *6* | *6* | *7* | *8* | *6* |
| Na^+^ (mmol l^-1^) | 148±1 | | 145±2 | | 148±2 | | 149±4 | | 148±2 | | 148±2 | | 147±2 | | 149±1 | |
|  | 146±1 | 150±2 | 143±3 | 148±4 | 145±2 | 152±2 | 151±6 | 146±5 | 145±2 | 151±1 | 146±1.4 | 150±4 | 143±3 | 149±4 | 147±2 | 150±2 |
| *N, treatment groups* | *14* | | *15* | | *15* | | *9* | | *14* | | *14* | | *13* | | *14* | |
| *N, sexes* | *8* | *6* | *9* | *6* | *8* | *7* | *5* | *4* | *7* | *7* | *8* | *6* | *6* | *7* | *8* | *6* |
| K^+^ (mmol l^-1^) | 2.9±0.3 | | 2.6±0.1 | | 2.6±0.1 | | 2.7±0.4 | | 3.6±0.3 | | 2.8±0.2 | | 3.2±0.1 | | 2.8±0.1 | |
|  | 2.7±0.3 | 3.1±0.5 | 2.5±0.2 | 2.8±0.2 | 2.7±0.4 | 2.9±0.2 | 2.5±0.5 | 3.0±0.6 | 3.3±0.2 | 3.8±0.4 | 2.5±0.1 | 3.2±0.4 | 3.3±0.2 | 4.4±0.9 | 2.7±0.2 | 2.9±0.2 |
| *N, treatment groups* | *14* | | *15* | | *15* | | *9* | | *14* | | *14* | | *13* | | *14* | |
| *N, sexes* | *8* | *6* | *9* | *6* | *8* | *7* | *5* | *4* | *7* | *7* | *8* | *6* | *6* | *7* | *8* | *6* |
| Glucose (mmol l^-1^) | 6.0±0.4 | | 7.4±1.1 | | 5.9±0.4 | | 6.9±1.3 | | 6.1±0.3 | | 6.8±0.7 | | 6.9±0.3 | | 7.0±0.6 | |
|  | 6.4±0.5 | 5.4±0.7 | 8.7±1.7 | 5.5±0.2 | 6.3±0.5 | 5.3±0.5 | 8.5±2.2 | 5.0±0.2 | 7.5±0.5 | 5.6±0.4 | 7.3±1.1 | 6.1±0.6 | 7.5±0.5 | 6.3±0.4 | 7.3±1.0 | 6.7±0.5 |
| *N, treatment groups* | *14* | | *15* | | *15* | | *9* | | *14* | | *14* | | *13* | | *14* | |
| *N, sexes* | *8* | *6* | *9* | *6* | *8* | *7* | *5* | *4* | *7* | *7* | *8* | *6* | *6* | *7* | *8* | *6* |
| Cortisol (ng l^-1^) | 102±38 | | 70±20 | | 146±32 | | 208±52 | | 252±40 | | 276±57 | | 209±47 | | 123±26 | |
|  | 156±61 | 31±8 | 101±29 | 23±5 | 235±37 | 45±10 | 316±54 | 74±22 | 379±37 | 125±16 | 384±80 | 133±19 | 363±49 | 77.0±18 | 172±33 | 54±12 |
| *N, treatment groups* | *14* | | *15* | | *15* | | *9* | | *14* | | *14* | | *13* | | *14* | |
| *N, sexes* | *8* | *6* | *9* | *6* | *8* | *7* | *5* | *4* | *7* | *7* | *8* | *6* | *6* | *7* | *8* | *6* |

*Values (mean±SEM) for all individuals within each treatment group (centered values on top of each variable row) are presented, as well as the values and sample sizes (n) for the treatment groups and female (F) and male (M) fish within each treatment group. Statistical details are presented in Figure 3 and Table S3. P_V_O_2_ = Partial pressure of venous oxygen, MCHC= Mean corpuscular [haemoglobin].*

| Table S3. Full disclosure of the statistical details from the mixed model analyses performed on the data illustrated in figure 3. | | | | |
| --- | --- | --- | --- | --- |
|  | P_V_O_2_ | Haematocrit | [Haemoglobin] | Mean corpuscular [Haemoglobin] |
| Level | ***F_3,59_*=31.4; *P*>0.001** | ***F_3,64_*=4.9; *P=*0.004** | *F_3,64_*=0.9; *P=*0.46 | ***F_3,67_*=4.8; *P=*0.004** |
| Treatment | *F_1,31_*=2.7; *P*=0.11 | *F_1,30_*=1.3; *P*=0.26 | *F_1,29_*=0.3; *P*=0.58 | *F_1,34_*=0.8; *P*=0.37 |
| Sex | *F_1,31_*=2.8; *P=*0.11 | *F_1,30_*=1.8; *P*=0.19 | *F_1,29_*=2.0; *P*=0.17 | *F_1,34_*=0.0; *P*=0.94 |
| Level*Treatment | *F_3,59_*=0.8; *P=*0.52 | *F_3,64_*=1.1; *P*=0.34 | *F_3,64_*=1.7; *P*=0.18 | *F_3,67_*=0.8; *P*=0.50 |
| Level*Sex | *F_3,59_*=0.9; *P=*0.46 | *F_3,64_*=2.5; *P*=0.07 | *F_3,64_*=1.3; *P*=0.28 | *F_3,67_*=1.3; *P*=0.27 |
| Treatment*Sex | *F_1,31_*=3.3; *P=*0.08 | *F_1,30_*=0.2; *P*=0.68 | *F_1,29_*=0.3; *P*=0.59 | *F_1,34_*=1.5; *P*=0.24 |
| Level*Treatment*Sex | ***F_3,59_*=3.0; *P=*0.04** | *F_3,64_*=1.5; *P*=0.22 | *F_3,64_*=0.8; *P*=0.51 | *F_3,67_*=0.5; *P*=0.68 |
| *Statistically significant (P<0.05) model outcomes are depicted in bold. P_V_O_2_ = Partial pressure of venous oxygen.* | | | | |

| Table S4. Full disclosure of the statistical details from the mixed model analyses performed on the data illustrated in figure 4. | | | | | |
| --- | --- | --- | --- | --- | --- |
|  | [Lactate] | [Na^+^] | [K^+^] | [Glucose] | [Cortisol] |
| Level | ***F*_3,57_=42.6; *P*>0.001** | *F*_3,59_=1.0; *P*=0.38 | ***F_1,59_*=7.8; *P*>0.001** | ***F*_3,57_=42.6; *P*>0.001** | ***F_3,60_*=31.0; *P*>0.001** |
| Treatment | *F_1_*_,27_=4.0; *P*=0.056 | *F*_1,26_=0.0; *P*=0.98 | ***F_1,31_*=6.9; *P*=0.013** | *F*_1,27_=4.0; *P=*0.06 | *F_1,28_*=0.7; *P*=0.43 |
| Sex | ***F_1_*_,27_=5.5; *P=*0.027** | ***F*_1,26_=4.9; *P=*0.04** | *F_3,31_*=3.4; *P=*0.07 | ***F*_1,27_ =5.5; *P*=0.03** | ***F_1,28_*=67.9;** ***P*>0.001** |
| Level*Treatment | *F*_3,57_=2.2; *P*=0.10 | *F*_3,59_=0.9; *P*=0.44 | *F_3,59_*=0.9; *P*=0.47 | *F*_3,57_=2.2; *P*=0.10 | *F_3,60_*=2.0; *P*=0.13 |
| Level*Sex | *F*_3,57_=0.7; *P=*0.53 | *F*_3,59_=0.2; *P*=0.87 | *F_3,59_*=1.6; *P*=0.19 | *F*_3,57_=0.7; *P*=0.53 | *F_3,60_*=1.8; *P=*0.16 |
| Treatment*Sex | *F_1_*_,27_=0.2; *P*=0.67 | *F*_1,26_=0.6; *P*=0.44 | *F_1,31_*=0.4; *P*=0.55 | *F*_1,27_=0.2; *P*=0.67 | *F_1,28_*=0.3; *P*=0.58 |
| Level*Treatment*Sex | *F*_3,57_=0.1; *P*=0.94 | *F*_3,59_=0.6; *P*=0.62 | *F_3,59_*=0.3; *P*=0.83 | *F*_3,57_=0.1; *P*=0.94 | *F_3,60_*=0.4; *P*=0.75 |
| *Statistically significant (P<0.05) model outcomes are depicted in bold.* | | | | | |

| Table S5. Sample sizes for the data illustrated in figure 5. | | | | | | | | | | | | |
| --- | --- | --- | --- | --- | --- | --- | --- | --- | --- | --- | --- | --- |
| *Temperature (°C)* | | 8 | | 15 | | 20 | | 25 | | 30 | |  |
|  | | Sham operated | Coronary-ligated | Sham operated | Coronary-ligated | Sham operated | Coronary-ligated | Sham operated | Coronary-ligated | Sham operated | Coronary-ligated |  |
| Cardiac LDH | *Treatment groups* | 14 | 14 | 14 | 14 | 14 | 14 | 14 | 14 | 14 | 14 |  |
|  | *Female/male* | 7/7 | 7/7 | 7/7 | 7/7 | 7/7 | 7/7 | 7/7 | 7/7 | 7/7 | 7/7 |  |
| White muscle LDH | *Treatment groups* | 15 | 14 | 15 | 14 | 15 | 14 | 15 | 14 | 15 | 14 |  |
|  | *Female/male* | 8/7 | 7/7 | 8/7 | 7/7 | 8/7 | 7/7 | 8/7 | 7/7 | 8/7 | 7/7 |  |
| Cardiac CS | *Treatment groups* | 14 | 14 | 14 | 14 | 14 | 14 | 14 | 14 | 12 | 9 |  |
|  | *Female/male* | 7/7 | 7/7 | 7/7 | 7/7 | 7/7 | 7/7 | 7/7 | 7/7 | 6/6 | 6/3 |  |
| Red muscle CS | *Treatment groups* | 15 | 14 | 15 | 14 | 15 | 14 | 15 | 14 | 9 | 12 |  |
|  | *Female/male* | 8/7 | 7/7 | 8/7 | 7/7 | 8/7 | 7/7 | 8/7 | 7/7 | 5/4 | 6/6 |  |

Abbreviations are: LDH = Lactate dehydrogenase, CS = Citrate Synthase

| Table S6. Full disclosure of the statistical details from the mixed model analyses performed on the data illustrated in figure 5. | | | | |
| --- | --- | --- | --- | --- |
|  | Cardiac LDH | White muscle LDH | Cardiac CS | Red muscle CS |
| Temperature | ***F*_4,95_=33.0; *P*>0.001** | ***F*_4,100_=293.6; *P*>0.001** | ***F_4,86_*=67.9; *P*>0.001** | ***F*_4,92_=511.7; *P*>0.001** |
| Treatment | ***F*_1,25_=860.5; *P*>0.001** | *F*_1,27_=0.1; *P*=0.80 | ***F_1,25_*=15.9; *P*=0.001** | *F*_1,25_=1.0; *P=*0.34 |
| Sex | *F_1_*_,25_=1.5; *P=*0.24 | *F*_1,27_=0.7; *P=*0.40 | *F_1,25_*=0.1; *P=*0.72 | *F*_1,25_ =0.1; *P*=0.76 |
| Temperature*Treatment | ***F*_4,95_=4.3; *P*=0.003** | *F*_4,100_=0.9; *P*=0.48 | *F_4,86_*=1.1; *P*=0.38 | *F*_4,92_=1.9; *P*=0.11 |
| Temperature*Sex | ***F*_4,95_=3.2; *P*=0.02** | *F*_4,100_=0.7; *P*=0.59 | *F_4,86_*=0.4; *P*=0.85 | *F*_4,92_=0.3; *P*=0.91 |
| Treatment*Sex | *F_1_*_,25_=2.0; *P*=0.17 | *F*_1,27_=0.07; *P*=0.79 | *F_1,25_*=2.8; *P*=0.11 | *F*_1,25_=0.5; *P*=0.49 |
| Temperature*Treatment*Sex | *F*_4,95_=0.7; *P*=0.62 | *F*_4,100_=0.4; *P*=0.80 | *F_4,86_*=0.6; *P*=0.68 | *F*_4,92_=0.6; *P*=0.69 |
| *Statistically significant (P<0.05) model outcomes are depicted in bold. LDH = Lactate dehydrogenase; CS=Citrate synthase.* | | | | |
